# Global diversity and balancing selection of 23 leading *Plasmodium falciparum* candidate vaccine antigens

**DOI:** 10.1371/journal.pcbi.1009801

**Published:** 2022-02-02

**Authors:** Myo T. Naung, Elijah Martin, Jacob Munro, Somya Mehra, Andrew J. Guy, Moses Laman, G. L. Abby Harrison, Livingstone Tavul, Manuel Hetzel, Dominic Kwiatkowski, Ivo Mueller, Melanie Bahlo, Alyssa E. Barry

**Affiliations:** 1 Population Health and Immunity Division, Walter and Eliza Hall Institute of Medical Research, Parkville, Victoria, Australia; 2 Department of Medical Biology, University of Melbourne, Carlton, Victoria, Australia; 3 Institute of Mental and Physical Health and Clinical Translation (IMPACT), School of Medicine, Deakin University, Geelong, Victoria, Australia; 4 Life Sciences Discipline, Burnet Institute, Melbourne, Victoria, Australia; 5 School of Science, RMIT University, Melbourne, Victoria, Australia; 6 Vector Borne Diseases Unit, Papua New Guinea Institute of Medical Research, Madang, Papua New Guinea; 7 Swiss Tropical Public Health Institute, Basel, Switzerland; 8 University of Basel, Basel, Switzerland; 9 Sanger Institute, Hinxton, United Kingdom; 10 Big Data Institute, University of Oxford, Oxford, United Kingdom; 11 Division of Parasites and Insect Vectors, Pasteur Institute, Paris, France; US Army Medical Research and Materiel Command: US Army Medical Research and Development Command, UNITED STATES

## Abstract

Investigation of the diversity of malaria parasite antigens can help prioritize and validate them as vaccine candidates and identify the most common variants for inclusion in vaccine formulations. Studies of vaccine candidates of the most virulent human malaria parasite, *Plasmodium falciparum*, have focused on a handful of well-known antigens, while several others have never been studied. Here we examine the global diversity and population structure of leading vaccine candidate antigens of *P*. *falciparum* using the MalariaGEN Pf3K (version 5.1) resource, comprising more than 2600 genomes from 15 malaria endemic countries. A stringent variant calling pipeline was used to extract high quality antigen gene ‘haplotypes’ from the global dataset and a new R-package named *VaxPack* was used to streamline population genetic analyses. In addition, a newly developed algorithm that enables spatial averaging of selection pressure on 3D protein structures was applied to the dataset. We analysed the genes encoding 23 leading and novel candidate malaria vaccine antigens including *csp*, *trap*, *eba175*, *ama1*, *rh5*, and *CelTOS*. Our analysis shows that current malaria vaccine formulations are based on rare haplotypes and thus may have limited efficacy against natural parasite populations. High levels of diversity with evidence of balancing selection was detected for most of the erythrocytic and pre-erythrocytic antigens. Measures of natural selection were then mapped to 3D protein structures to predict targets of functional antibodies. For some antigens, geographical variation in the intensity and distribution of these signals on the 3D structure suggests adaptation to different human host or mosquito vector populations. This study provides an essential framework for the diversity of *P*. *falciparum* antigens to be considered in the design of the next generation of malaria vaccines.

## Author summary

Highly effective malaria vaccines are important for the sustainable elimination of malaria. However, the diversity of parasite antigens targeted by malaria vaccines has been largely overlooked, with most vaccine formulations based only on a single antigen variant. Failure to accommodate this diversity may result in vaccines only being effective against vaccine-like variants, resulting in limited protective efficacy. Investigation of the diversity of genes encoding parasite antigens can help prioritize and validate them as vaccine candidates as well as to identify the most common variants for inclusion in the next generation of malaria vaccines. Here we measure the diversity of 23 vaccine antigens of *Plasmodium falciparum*, using the publicly available MalariaGEN Pf3K (version 5.1) resource comprising more than 2600 genomes from 15 malaria endemic countries. We find that variants found in current vaccine formulations are rare and thus may target only a small proportion of circulating malaria parasite strains. Variation in intensity of immune selection in parasites from different geographic areas suggests adaptation to different human host or vector populations. This study provides an essential framework for the design of the next generation of malaria vaccines, in addition to providing novel insights into malaria biology.

## Introduction

*Plasmodium falciparum*, the most lethal human malaria parasite, has been co-evolving with its human host for tens of thousands of years [1]. Along this evolutionary timeline, host-parasite interactions have left landmarks of selection on both genomes [2,3]. These landmarks can help to identify parasite surface proteins targeted by host immune responses and therefore antigens that could be used in “subunit” vaccines to protect against infection and disease. However, malaria parasites evade host immune responses through the accumulation of mutations, thus increasing the repertoire of variants circulating in the parasite population, and therefore this diversity needs to be considered in vaccine formulations [4,5]. Otherwise, vaccines may only be partially effective resulting in a “sieve effect”, with higher efficacy against vaccine-like strains and low to no efficacy against antigenically distinct strains [6,7,8]. Following vaccination, vaccine-distinct variants might become dominant, requiring new vaccine formulations to be developed. One approach to overcome parasite diversity is to include multiple variants currently circulating in the global population, as is the case for the influenza vaccine which is reformulated annually [9]. Malaria differs from the influenza virus however as malaria parasites have multiple lifecycle stages, hundreds of surface antigens that could be considered as vaccine candidates, and a lack of understanding of how parasite antigen diversity influences host immune responses. With the availability of thousands of malaria parasite genomes [10], characterisation of antigen diversity on a global scale is highly feasible and a crucial step in contemporary malaria vaccine development. A meta-population genetic analysis of antigens would provide a catalogue of common variants to be included in vaccine formulations and provide a basis for predicting vaccine effectiveness in different populations.

Malaria antigen diversity has been largely overlooked with most subunit vaccines based on a single antigen variant, predominantly those of the reference strain 3D7. As a result, very few have shown significant protective efficacy in human clinical trials, and protection is mostly short-lived. Of the numerous vaccine candidates that have been tested in clinical trials, only RTS, S, has completed Phase III clinical trials. RTS,S is based on the C-terminal and NANP repeat region of the Circumsporozoite Protein (CSP) of the African strain 3D7 [6,11]. The limited efficacy of this vaccine [12] has been attributed to polymorphism of the target antigen, PfCSP, suggesting a diversity-covering approach could be more effective [6,7,13]. This is supported by a study in Africa where the one year cumulative vaccine efficacy against homologous strains was 50.3% compared to 33.4% against heterologous strains [4]. Furthermore, in a phase 2b trial in Papua New Guinean (PNG) children, the “Combination B” malaria vaccine (multiantigen vaccine, composed of MSP2, RESA and a fragment of MSP1) showed 62% protective efficacy against vaccine (3D7) like strains and limited efficacy against vaccine dissimilar strains [8]. Of the vaccine trials that have been powered to measure allele specific outcomes (comparing infection rates of vaccine versus non-vaccine variants in vaccinees and controls), several have identified variant-specific efficacy (reviewed in [14]). Vaccine development would therefore benefit from a better understanding of the global diversity and distribution of antigen variants, to select common variants that could maximise vaccine efficacy.

The presence of intermediate variant frequencies for a given antigen in a parasite population indicates balancing selection, which is driven by protective immune responses [15–17]. Balancing selection can be measured using the *Tajima’s D* statistic using a ‘sliding window’ approach to identify hotspots of selection, along the length of a gene [18]. However, standard Tajima’s D analyses of linear gene sequences do not consider the spatial distribution of polymorphic residues (i.e. residues that are distant on the linear sequence may be proximal on the three-dimensional (3D) structure). Despite the availability of high quality whole genome sequence data from thousands of worldwide *P*. *falciparum* isolates, there has been no population genetic analysis of malaria vaccine candidate antigens using this data, and only a few studies have mapped selection hotspots onto experimentally-determined or modelled 3D protein structures [16,17,19]. In addition, only a few studies have analysed the diversity of existing or emerging *P*. *falciparum* vaccine candidate antigens and the majority have focused on few antigens and geographic areas.

In this study, we aimed to conduct a meta-population genetic analysis to characterise the global genetic diversity of 23 emerging and established *P*. *falciparum* vaccine candidates from different life cycle stages and sub-cellular localizations. Antigens were included if they were mentioned in the WHO Malaria Rainbow Table and in previous pre-clinical or clinical vaccine development studies [2,6,20]. They include antigens that have well-defined three dimensional (3D) structures as well as disordered proteins that are not well characterised [21]. The analysis was based on *P*. *falciparum* WGS from the Pf3K (version 5.1) global data resource [10] which comprises more than 2,600 WGS from 15 countries and 156 additional WGS from PNG [22,23] generated in collaboration with the MalariaGEN *P*. *falciparum* Community Project. These comprehensive population genetic analyses of malaria vaccine candidates will aid the development of more broadly effective vaccines.

## Results

WGS data from a total of 2661 *P*. *falciparum* isolates from 16 countries from the Asia-Pacific and Africa were processed to obtain gene sequences for population genetic analysis. The pipeline filters the dataset to remove high complexity infections as determined by *F*_ws_ [24]. This step aimed to ensure the correct assignment of variants to a single major clone within the sample (S2 Fig). We found a large proportion (greater than 30%) of field isolates from African regions predicted to have more than two clones, especially in Malawi and Ghana, likely reflecting high transmission intensity in those areas. However, the number of genomes in the final dataset (n = 1499) remained high, even after removing these samples. From these genomes, gene sequences for 23 antigens (Table 1) were obtained. This included a mean of 1374 (1079–1499) sequences per antigen, and 107 (26–433) sequences per country (Table 1, Table A in S1 Text). Nigeria was included in overall genetic diversity analyses but removed from further analyses due to very low sample size (n = 4).

**Table 1:** Vaccine Candidate Antigens. P. falciparum antigens and associated domains selected for the analysis were functionally important for malaria biology and key candidates of malaria vaccines trials.

Lifecycle Stage	Name	Gene ID	Genomic Length (bp)	Domains	3D Structure
**Pre-Erythrocytic**	*Csp*	PF3D7_0304600	1194	C-terminal	PDB Code: 3VDJ
	*Ctrp*	PF3D7_0315200	6345	Full-length	NA
	*Trap*	PF3D7_1335900	1725	Ectodomain	PDB Code: 4HQF.A
	*Exp1*	PF3D7_1121600	937	Full-length	NA
	*Starp*	PF3D7_0702300	1970	Full-length	NA
	*Glurp*	PF3D7_1035300	3702	Full-length	NA
	*Trep*	PF3D7_1442600	10632	Full-length	NA
**Erythrocytic**	*Ama1*	PF3D7_1133400	1869	I—III	Manually developed model [32]
	*Ron2*	PF3D7_1452000	6570	Full length	NA
	*Eba175*	PF3D7_0731500	4875	RII, RIII-V	Model based on 1ZRO template
	*Rh5*	PF3D7_0424100	1788	Full-length	PDB Code: 6MPV.B
	*Ripr*	PF3D7_0323400	3261	Full-length	NA
	*Cyrpa*	PF3D7_0423800	1188	Full-length	PDB Code: 6MPV.A
	*Msp1*	PF3D7_0930300	5163	*Msp1-19*	Model based on 1OB1 template
	*Msp3*	PF3D7_1035400	1065	Full-length	NA
	*Msp4*	PF3D7_0207000	963	Full-length	NA
	*Msp6*	PF3D7_1035500	1116	Full-length	NA
	*Ralp1*	PF3D7_0722200	2250	Full-length	NA
	*Resa*	PF3D7_0102200	3464	Full-length	NA
	*Sera5*	PF3D7_0207600	3435	C-terminal	PDB Code: 2WBF
	*Sera8*	PF3D7_0207300	2536	Full-length	NA
**Gametocyte**	*Tramp*	PF3D7_1218000	1059	Full-length	NA
	*Pfs48/45*	PF3D7_1346700	1347	Full-length, 6C	PDB Code: 6E62.A
	*Celtos*	PF3D7_1216600	549	Full-length	Model Based on 5TSZ template

### High haplotype diversity and balancing selection indicates antigens that are natural targets of host immunity

**Fig 1:**
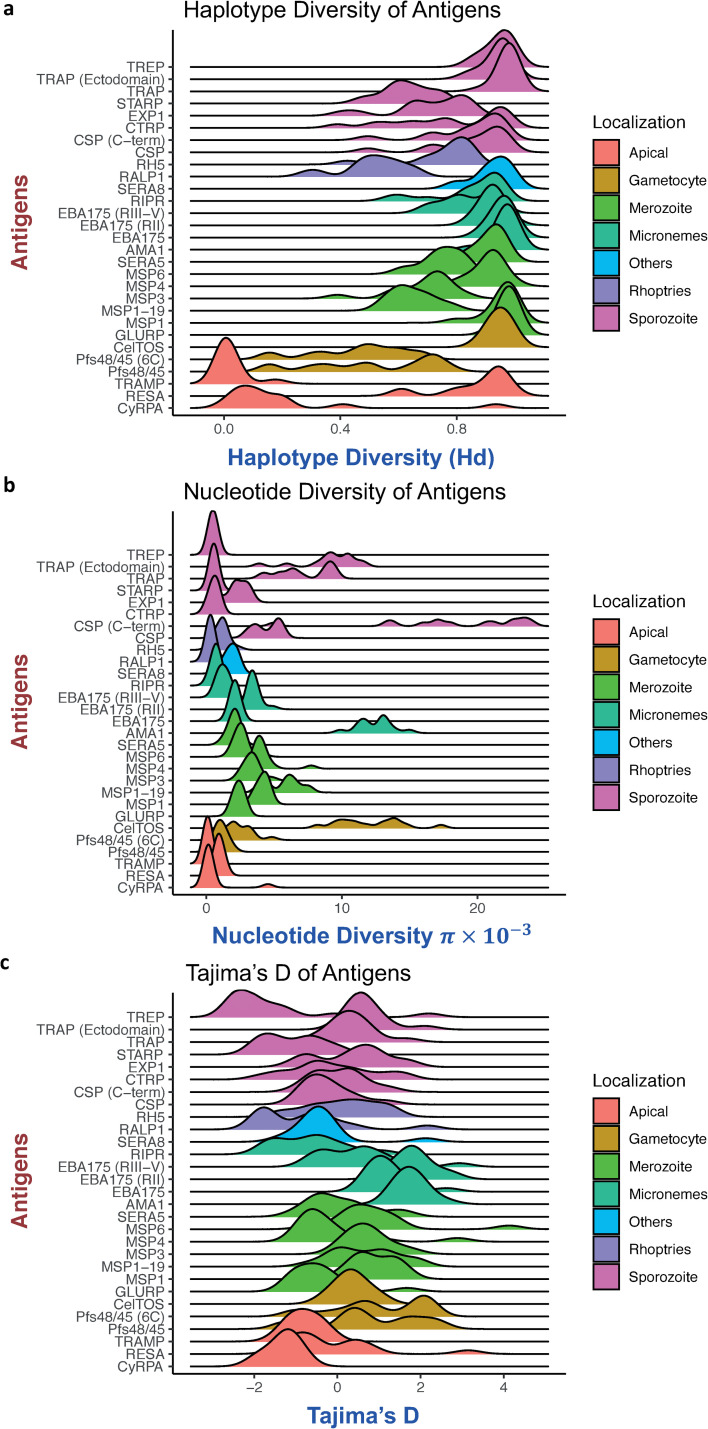
Distribution of haplotype diversity, nucleotide diversity, and Tajima’s D values amongst countries for each antigen. The lines from ridgeline plots indicate the range and distribution of respective diversity parameters for each antigen across different parasite populations (countries). Tramp, and cyrpa were conserved across all populations, whilst trap, ama1, eba175, and celtos were diverse and showed evidence of diversifying selection across all parasite populations.

Overall genetic diversity analyses based on Nei’s nucleotide diversity (π), haplotype diversity and Tajima’s D analyses indicated high proportions of SNPs that were non-synonymous (dN) and regions of balancing selection in most antigens [25] (Table A in S1 Text, Fig 1). In addition, the antigen subdomains, *csp*-C-terminal, *eba175* -RII and *pfs48/45* -6C were as diverse as the respective full-length gene (Fig 1, Table A in S1 Text). Different patterns of diversity across parasite populations (defined as all samples from a particular country) were observed for the antigens. For instance, *trap* had high haplotype diversity yet low to moderate nucleotide diversity indicating that many haplotypes were the result of very rare polymorphisms (Fig 1, Table A in S1 text). Whereas *csp* had high haplotype diversity and high (albeit variable amongst populations) nucleotide diversity, which might be related to differences in transmission intensity in different countries (Fig 1). In addition, *celtos* (a gametocyte antigen) also had high haplotype diversity but moderate to high (and variable) nucleotide diversity, which may indicate adaptation to mosquito vectors from different geographical regions (Fig 1, Table A in S1 Text). Moderate haplotype diversity, but low nucleotide diversity for *rh5* indicates lower diversity among distributed *rh5* haplotypes (Fig 1, Table A in S1 Text).

Genetic diversity and evidence of diversifying selection on the antigens was not related to their subcellular localization [26] (Fig 1). However, the diversity of some antigens (e.g. CSP) varies by geographic origin indicating that transmission intensity or other location-specific factors may play a role as previously shown in Barry *et al*. 2009 [15].

The use of full-length or full-domain *Tajima’s D* values can mask variable patterns within a gene when there are discrete stretches of positive *D* values linked by regions with neutral or highly negative D values [27]. Further analyses were therefore performed on these antigens for full-length or their functionally important domains.

### Antigens under balancing selection have large numbers of low frequency non-3D7 haplotypes

We identified the relationships amongst non-synonymous SNP haplotypes using network analysis for 17 of the 23 antigens. Six antigens were excluded because of lack of diversity. Many rare haplotypes are present within African populations, and relatively common haplotypes within Asia-Pacific countries, most likely owing to higher transmission in Africa. Generally, for antigens under balancing selection such as CelTOS, TRAP, AMA1, EBA175, MSP1 and GLURP (Fig 1), high haplotype diversity (Hd > 0.97) was ubiquitous amongst countries with no predominant haplotype (Table B in S1 Text). The majority of these are blood stage antigens suggesting they are dominant targets of natural host immunity.

**Fig 2:**
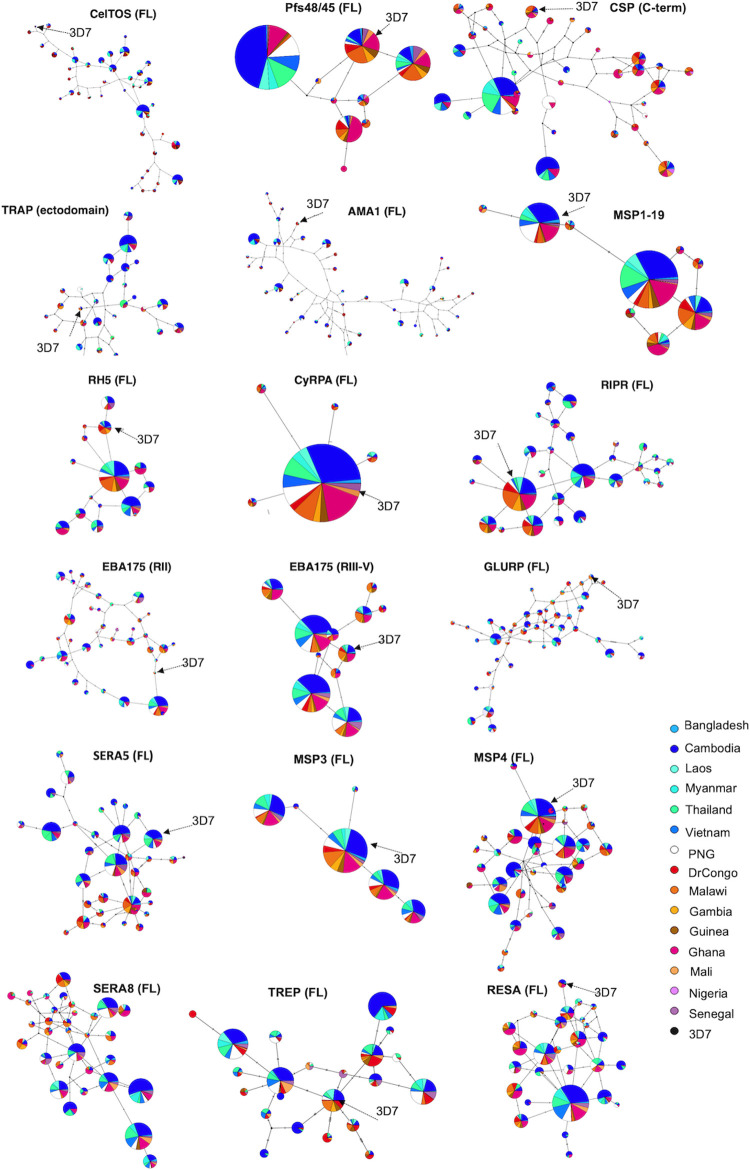
Haplotype network for malaria vaccine antigens. Templeton, Crandall, and Sing (TCS) network summarizing the global diversity of selected antigens using only common haplotypes (> 0.5% of all haplotypes) based on non-synonymous SNPs for full length respective domain of each antigen. Circles represent unique haplotypes, and circles are scaled according to the prevalence of the observed haplotypes. The number of non-synonymous SNP differences between each haplotype was shown by the number of hatch marks on the branches. The vaccine strain 3D7 (arrowed) was included for reference.

Haplotypes were often found in many countries (i.e. multiple colours for each node) suggesting minimal geographic variation (Fig 2). However, geographical clustering of haplotypes was observed for CTRP, TRAP (ectodomain), CSP (C-term), GLURP, EBA175 (RII), CelTOS, and Pfs48/45. Haplotypes from the same geographical region (e.g. Africa) were more closely related than those from different regions (e.g. Africa versus Asia, Fig 2). Most of these antigens are pre-erythrocytic or gametocyte antigens, consistent with previous observations [15] and may reflect adaptation of the parasite to local human and mosquito hosts.

Of all analysed antigens, the 3D7 haplotype was dominated only within CyRPA, MSP1-19, MSP3, MSP4, Pfs48/45, RALP1, RH5, RIPR, STARP, TRAMP, and TREP antigens (Fig 2, Tables A and B in S1 Text). For most antigens with multiple balancing selection hotspots such as AMA1, CSP (C-term), EBA175 (RII), CelTOS, the most common haplotypes were only remotely related to 3D7 vaccine haplotype (Table B in S1 Text). No 3D7 haplotypes were observed in the dataset for full length EBA175, MSP1 and SERA8 (Table A in S1 Text, Fig 2). This suggests that a 3D7-based malaria vaccine may be effective against only a small proportion of natural parasite populations.

### Polymorphic resides are surface exposed

**Fig 3:**
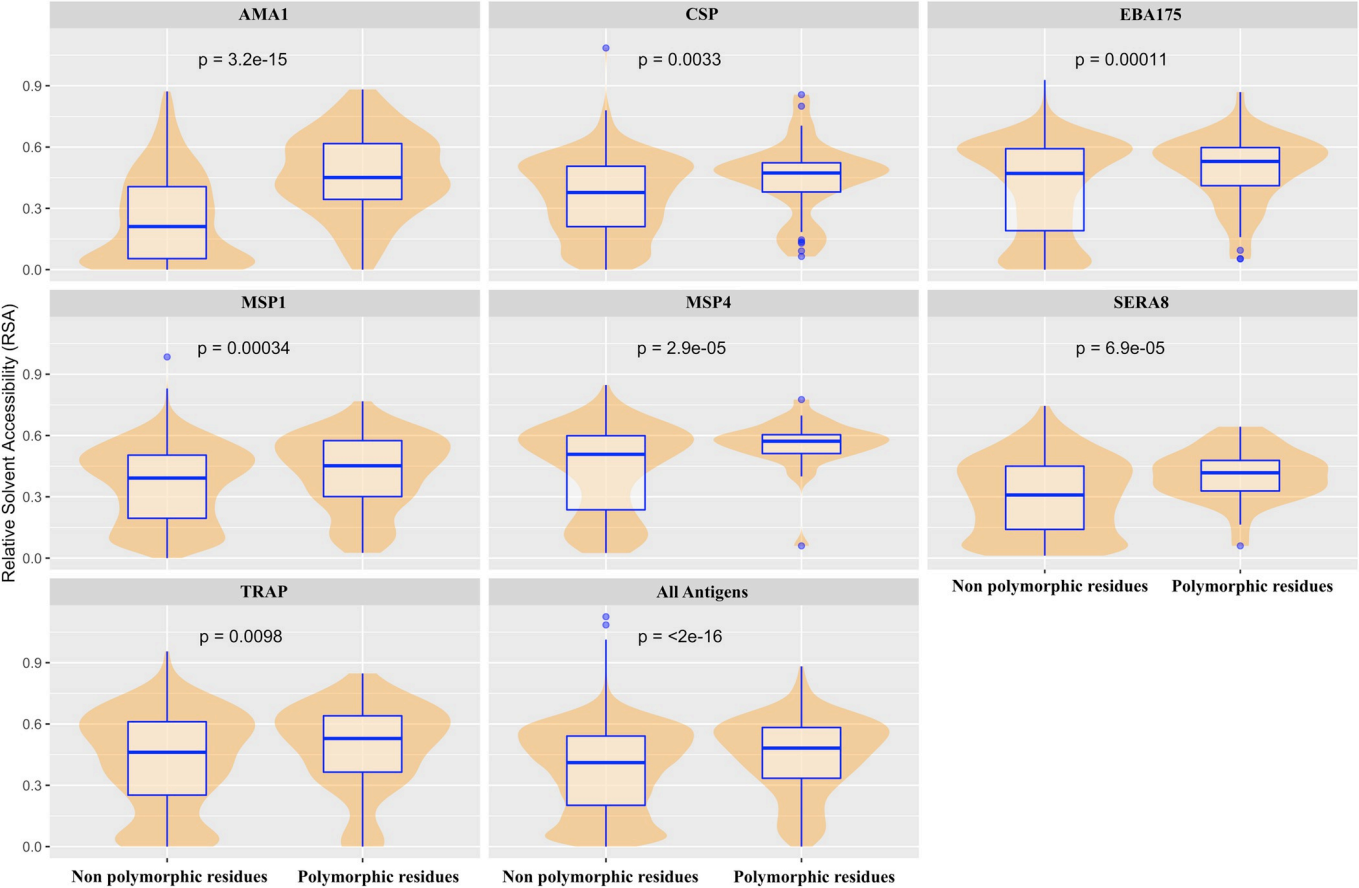
Relative solvent accessibility of polymorphic versus conserved residues. Relative solvent accessibility (RSA) was calculated for all residues for 23 antigens. RSA was calculated using neural network based NetSurfP1.1 program or DSSP program respectively based on the presence of known PDB or homology-modelled structures [30,31]. Polymorphic residues from more than 1000 sequences regardless of minor allele frequency (MAF) were included in the analysis. Box and whisker plots show the median (blue line), and interquartile range (blue box) of RSA values for each residue from respective group. The violin plot (which uses Kernel Density Estimation to compute an empirical probability distribution) shows a smooth distribution of RSA values for most of the calculated group. RSA scores for individual antigens as well as for the combination of all antigens were calculated. Only significant p-values are shown.

Relative solvent accessibility (RSA) analysis gives an indication of the regions of a protein that are exposed to the extracellular environment, and thus may be targeted by immune responses [16,28,29]. The RSA of all 23 antigens was compared for 792 polymorphic and 14,789 conserved residues. A higher RSA score suggests presence of a particular amino acid on the surface and thus making it able to interact with the host environment. Overall, when combining all antigens, most of the polymorphic residues had significantly higher mean RSA scores of 0.46 than conserved residues with a mean RSA score of 0.38 (p value < 2.0 * 10^−16^, t-test) (Fig 3). When individual antigens were analysed, only AMA1, CSP, EBA175, MSP1, MSP4, SERA8, and TRAP had significantly higher RSA scores than that of their residues without underlying polymorphisms (p < 0.05, t-test).

### Polymorphic residues are predominantly found at functionally important interfaces

**Fig 4:**
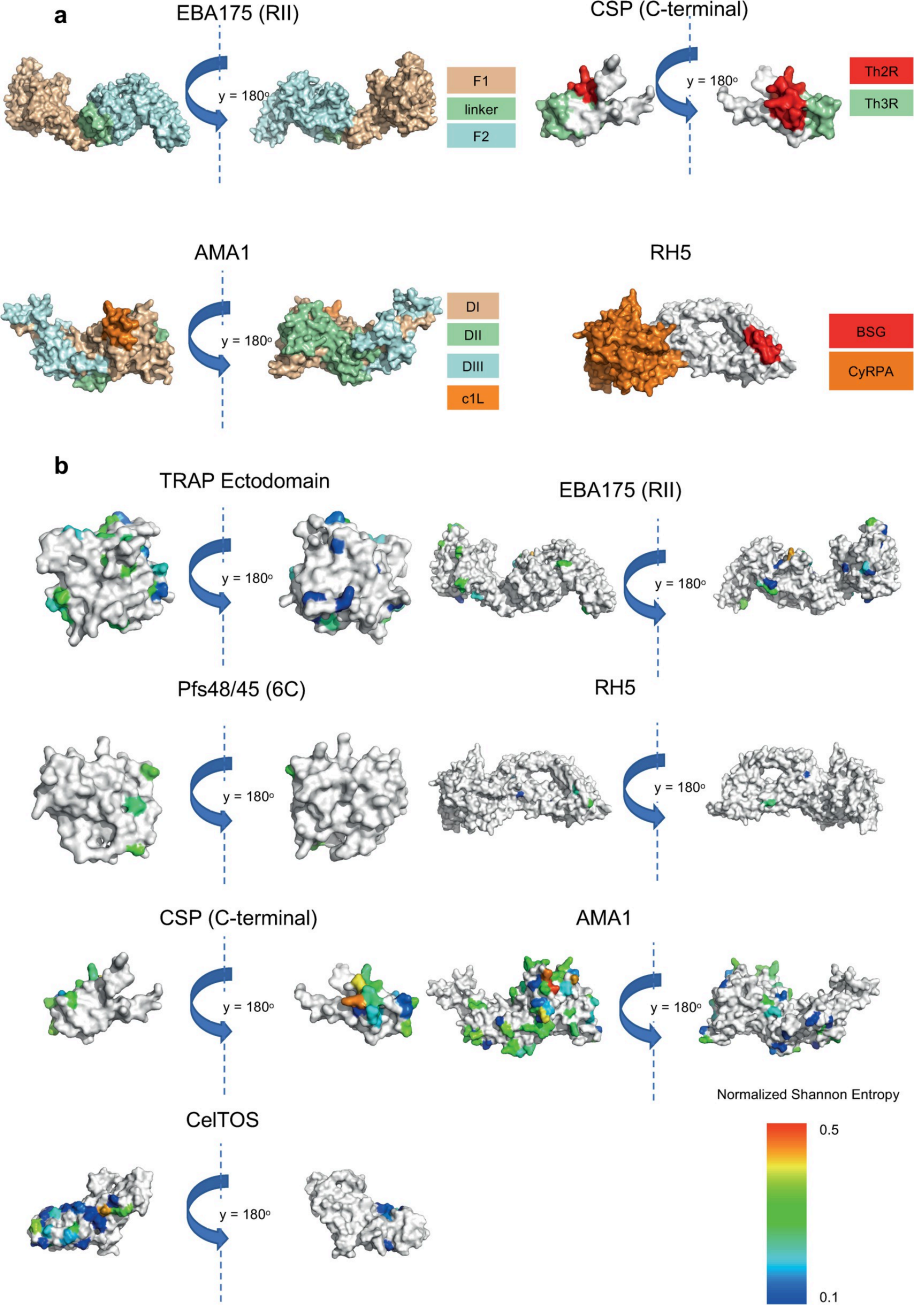
Mutations at functionally important interfaces. **(a)** Available domain and epitope information for AMA1, EBA175 (RII), RH5, and CSP (C-terminal). **(b)** Site specific diversity measure for CelTOS, Pfs48/45 (6C domain), TRAP (ectodomain), CSP (C-terminal), AMA1, EBA175 (RII) and RH5. Normalized Shannon Entropy was calculated per residue for these antigens (unavailable residues were coloured in white). Higher entropy values indicate higher diversity across all populations for a particular residue. Residues from the CSP Th2R epitope and AMA1 C1L loop have the highest entropy values. Very low entropy values across all populations were observed for SERA5, and CyRPA.

Shannon Entropy is a measure of the variability of individual amino acid residues within a protein. We calculated the normalized Shannon Entropy using all available sequences for each antigen that had an available 3D protein structure (Table 2) to investigate the potential functional impact of site-specific diversity. Except for CyRPA, SERA5 and MSP1-19, the normalized Shannon Entropy scores were high (>0.2) among the residues situated at host-receptor binding interface, within previously described immunological epitopes or under balancing selection (Fig 4, Table 2). For instance, residues situated at the surface exposed c1L loop of AMA1, and residues situated at the dimerization interface of EBA175 RII had high normalized Shannon Entropy scores. Mutations at functionally important interfaces may enable parasites to escape from host immune responses.

**Table 2:** Residues with Shannon Entropy Scores greater than 0.20 for each calculated antigen.

Antigens	Amino Acid Residuesa*
TRAP (ectodomain)	83, 90, 92, 98, 134
CSP (C-terminal)	318, 322, 352, 357
AMA1	182, 187, 190, 196, 197, 200, 201, 204, 225, 230, 242, 243, 267, 283, 300, 308, 404, 405, 439, 451, 485, 496, 503, 512
EBA175 (RII)	274, 279, 286, 388, 390, 584, 664
RH5	197
CyRPA	NA
SERA5	NA
MSP1-19	NA
Pfs48/45 (6C domain)	304, 322
CelTOS	318, 322, 352, 357

*Residue numbers were based on 3D7 sequence

### Evidence of balancing selection at functionally important interfaces for TRAP, AMA1, RIPR for all geographic populations but identification of geographically variable balancing selection hotspots for EBA175 (RII), RH5, CSP, CelTOS, and MSP1-19

An ideal malaria vaccine should be immunogenic and effective against naturally circulating strains from worldwide populations, but it is not feasible to include all haplotypes for each antigen we have described above. This is especially the case for highly diverse antigens such as CSP, TRAP, AMA1, EBA175, MSP1 (Table A in S1 Text). Therefore, for each antigen, it is important to identify the most immunologically relevant polymorphisms and gene regions, that are targets of host immunity and could influence vaccine efficacy. Typically, the *Tajima’s D* statistic has been calculated as a single metric encompassing the entire gene or domain of interest, or more recently, using a sliding window approach to identify hotspots of balancing selection along the length of a gene. Functional antibodies are critical for protective immunity from naturally acquired infection [6,32–37] however, more than 90% of functional antibody epitopes are discontinuous (non-linear) epitopes [38,39]. Thus, distant gene segments may be brought into proximity in three-dimensional (3D) protein structures. Consideration of structural features in 3D space are thus important, especially for antigens with highly surface exposed polymorphic residues such as AMA1, CSP, EBA175, and TRAP. Previous studies have suggested that polymorphic residues located on the surface of protein evolved to escape host immune responses [16,27,40]. We therefore examined selective pressure over the 3D protein structures of vaccine candidate antigens in the following analyses where possible.

A spatially derived approach to *Tajima’s D* (D*) was applied to available 3D structures (predicted or experimentally defined) for a subset of parasite populations covering all major endemic regions, including Southeast Asia, Africa, and Oceania (PNG). Structures included well-studied antigens like CSP (C-terminal), TRAP (ectodomain), AMA1, EBA175 (RII), MSP1, RH5, CyRPA, SERA5, CelTOS, and MSP1-19. We observed moderate to high D* scores (1.0–2.0), high scores (2.1–3.0), and extremely high scores (> 3.1) within most of these antigens, which is an indication of balancing selection focused on specific regions of the protein. Nearly neutral *Tajima’s D* values were found throughout the 3D structure of CyRPA, and SERA5 (C-terminal) for all parasite populations (S4 Fig). In general, we found a dichotomous pattern of balancing selection on some antigens for countries in the Asia-Pacific versus African populations.

**Fig 5:**
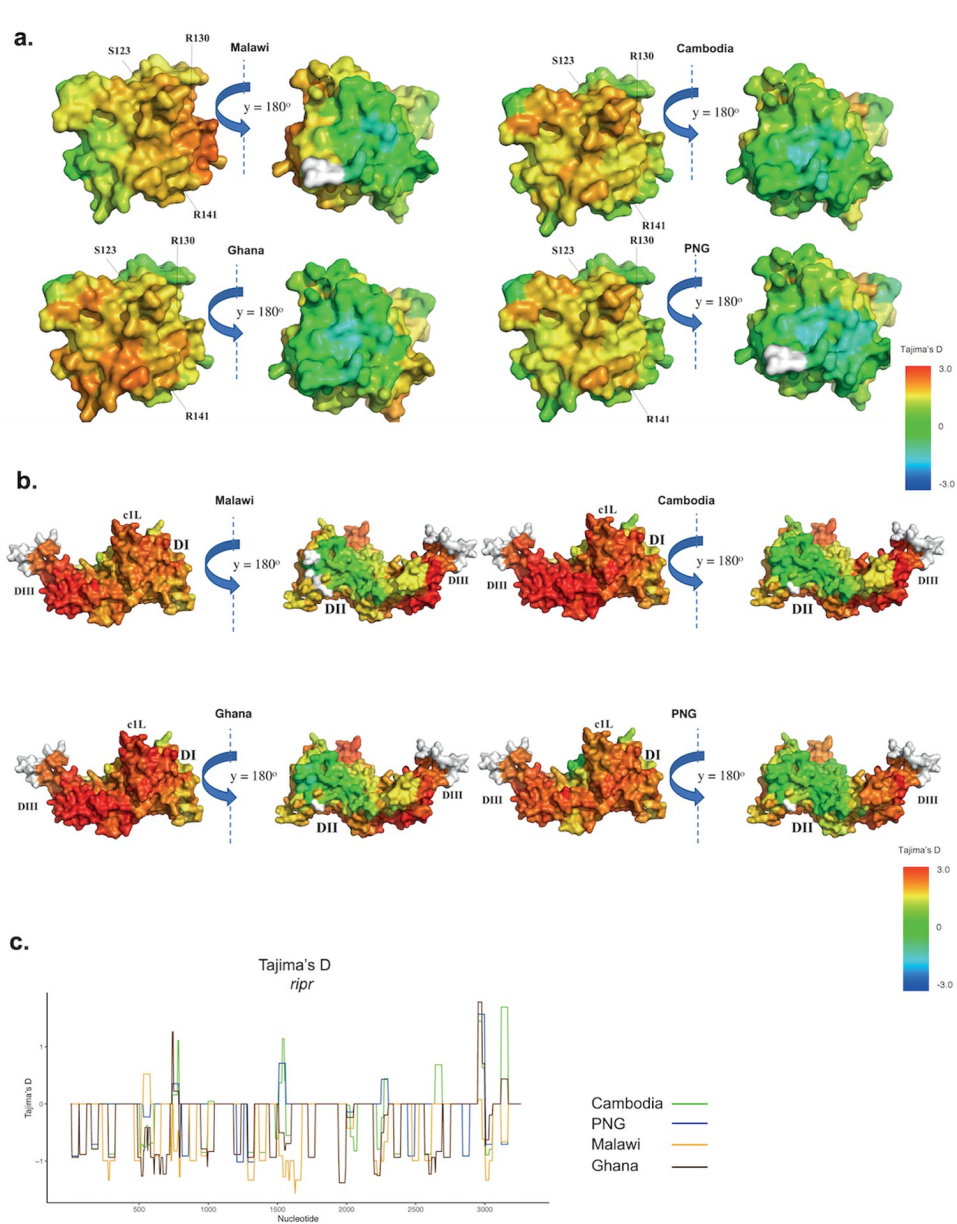
Antigens displaying geographically conserved balancing selection at functionally important interfaces. Antigens were not normalised based on their sizes. a. Spatially derived Tajima’s D (D*) score calculation for TRAP (Ectodomain) with incorporation of protein structural information using a 15Å window. TRAP (ectodomain) (PDB Code: 4HQF.A) was used. The structure was coloured according to D* scores mapped to each residue, and undefined D* are shown in white. Residues S123, R130 and R140 are involved in mediating heparin binding. Sample sizes: Malawi (n = 133), Ghana (n = 238), Cambodia (n = 430), and PNG (n = 112). b. Spatially derived Tajima’s D (D*) calculations for AMA1 with incorporation of protein structural information using 15 Å window. The manually modelled structure of AMA1 was used based on published results [27]. The structure was coloured according to D* scores mapped to each residue with undefined D* scores were shown in white. The DI, DII, DIII, and surface exposed c1L loop are indicated. Sample sizes: Malawi (n = 139), Ghana (n = 243), Cambodia (n = 433), and PNG (n = 112). c. Polymorphism and evidence of selection for ripr. Tajima’s D statistic calculated for disordered regions of RIPR in samples from Cambodia, PNG, Malawi, and Ghana. Tajima’s D is calculated with a sliding window approach (a window size of 50 bp and a step size of 5 bp). Nucleotide positions based on coding region are shown in the x-axis. Sample size for each respective population are as follows: Malawi (n = 137), Ghana (n = 246), Cambodia (n = 428), and PNG (n = 111).

The TRAP ectodomain (PDB Code: 4HQF.A) which is comprised of tandem von Willebrand factor A (VWA) and thrombospondin type I repeat (TSR) domains (AA residues: E41 –K240, 3D7 sequence) were included in our analyses [41]. In all populations, the suspected heparin binding interface of TRAP [42] has moderately higher *Tajima*’s D* scores than the opposite interface of the protein, which acts like the silent face of TRAP (ectodomain) (Fig 5A). Hence, residues Y89 –I100, and I115 –D146 showed moderate spatially derived D* scores (1.0–1.2) in all observed countries. Residues S123, T124, and N125 form a minor protrusion on the surface of TRAP, and residues R130, R141, K142 are thought to be mediated in heparin binding according to previous study [42]. However, within the active face of TRAP, the intensity of balancing selection (D* scores) appears to be geographically variable. Minimal nucleotide diversity variation was observed amongst geographical regions (Fig 1).

High D* scores (2.0–2.4) were found in all populations at the C1-L cluster [41,42] of AMA1 Domain I (AA residue: T194 –D212, 3D7 sequence), known to be associated with immune escape [16,43–45] (Fig 5B). Most of these residues were identified as discontinuous epitopes by the IEDB epitope prediction resource [46]. As expected, the entire AMA1 interface with hydrophobic binding cleft and RON2 interacting sites shows high balancing selection [47]. Similar to previous spatially derived analysis [16], the region on the border of DII and DIII domains (AA residues: P303 –F312, S432 –Y446, I479 –K508, 3D7 sequence) appears to have the highest D* scores (> 3) in all populations. This region was previously predicted as the surface exposed face of DII/DIII and suggests that these residues might be the targets of protective host immune responses within AMA1. A monoclonal antibody (1E10) against AMA1 DIII functions synergistically with antibodies against distant parts of AMA1 to inhibit merozoite growth [48]. This indicates that DIII of AMA1 plays a significant role as a target of functional antibody responses against *P*. *falciparum* in the context of natural infections. Consistent with previous findings [16,27,49], the spatially derived *Tajima’s D* values are unevenly distributed with high D* values mostly exclusive to one face of the AMA1 molecule, and close to zero on the opposing face of AMA1, which has been previously been described as the "silent face" [49,50] except in PNG where moderately high D* scores (1.9) were found at AA residues V524 –Y532. This is in line with the previous hypothesis that silent face of AMA1 has minimal exposure to the immune system [45,49]. The high level of spatial Tajima’s D* and nucleotide diversity was similar amongst populations for AMA1 suggesting that this antigen experiences similar selective pressures across different populations (Figs 5B, and S5).

*Ripr* was analysed in the context of the linear nucleotide sequence due to the poor predicted structure. The N-terminal region of RIPR contains 2 EGF-like domains, while the C-terminal region contains 8 EGF-like domains [51]. We found some degree of balancing selection (moderately high *D* values = 1.5) and high nucleotide diversity proximal to C-terminal EGF 7–10 region, located away from the CyRPA:RIPR interface [51] (nucleotide residues based on coding region 2900–2980, 3D7 sequence) of *ripr* at most of the populations (Fig 5C). These results were aligned with the anticipated RIPR-specific monoclonal antibody binding sites identified in a previous study [52]. This suggests the C-terminal region of RIPR is a target of host immunity in most populations.

**Fig 6:**
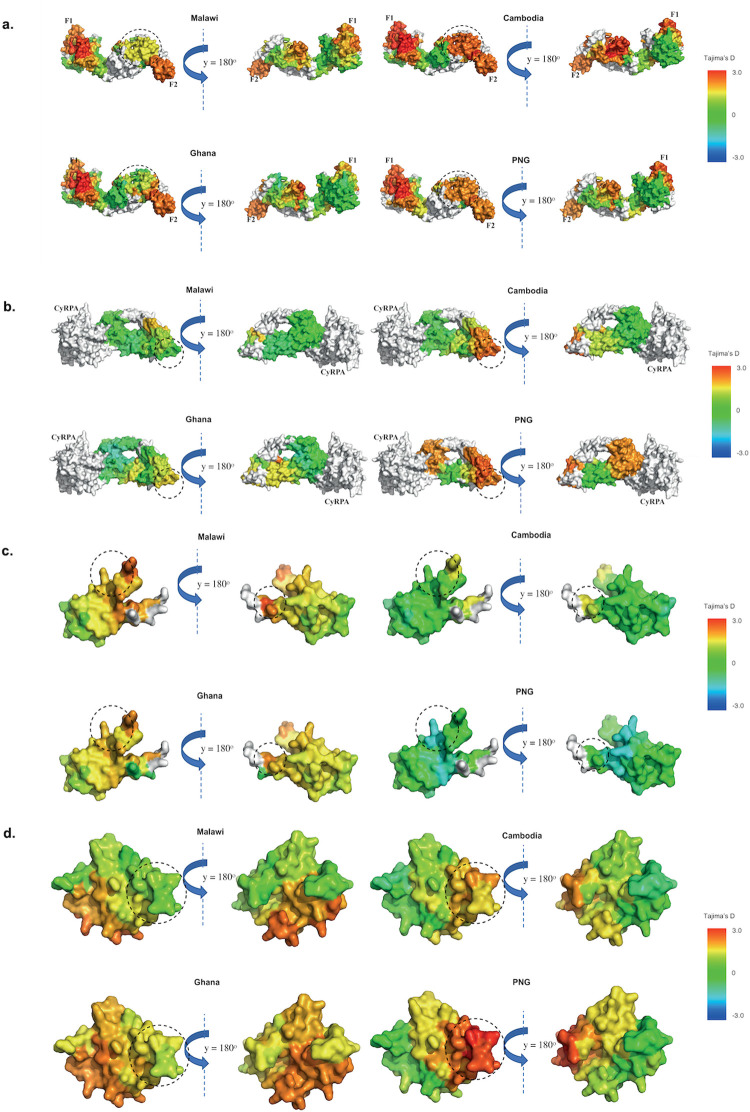
Antigens displaying geographically variable balancing selection hotspots. Antigens were not normalised based on their sizes. a. Spatially derived Tajima’s D (D*) for EBA175 with incorporation of protein structural information using 15 Å window. 3D7-based ModPipe model of EBA175 (RII) based on 1ZRO template was used. Structure was coloured according to D* scores mapped to each residue with undefined D* were shown in white. The highlighted region (in circle) shows different D* scores between Asia-Pacific and African countries. Sample sizes: Malawi (n = 136), Ghana (n = 237), Cambodia (n = 432), and PNG (n = 112). b. Spatially derived Tajima’s D (D*) calculation for countries from Asia-Pacific and Africa for RH5 with incorporation of protein structural information using 15Å window. Cryo-EM structure of RH5-CyRPA complex (PDB code: 6MPV.B) was used. Structure was coloured according to D* scores mapped to each residue with undefined D* and CyRPA were shown in white. The circle indicates Basigin-binding sites where different D* scores were observed between Asia-Pacific and African countries. Sample sizes: Malawi (n = 142), Ghana (n = 249), Cambodia (n = 433), and PNG (n = 112). c. Spatially derived Tajima’s D (D*) calculations for populations from Asia-Pacific and African regions for CSP (C-terminal) with incorporation of protein structural information using 15 Å window. The crystal structure of the thrombospondin receptor (TSR) domain of CSP [58] (PDB code: 3VDJ, chain A, AA residues: Y306—H376, 3D7 sequence), which consists of Th2R and Th3R (T-cell epitopes) was used [59]. Structure was coloured according to D* scores mapped to each residue with undefined D* were shown in white. The highlighted region (in circle) shows different D* scores between Asia-Pacific and African countries. Sample sizes: Malawi (n = 135), Ghana (n = 223), Cambodia (n = 431), and PNG (n = 111). d. Tajima’s D (D*) calculation for geographic area or countries from the Asia-Pacific and African regions for MSP1_19_ with incorporation of protein structural information using 15 Å window. The structured region of MSP1 (MSP1-19, AA residue: N1607—S1699) is based on the ModPipe homology model using template (PDB code: 1OB1) [60]. The structure was coloured according to D* scores mapped to each residue with undefined D* were shown in white. The circle indicates variable balancing selection hotspots between Asia-Pacific and African countries. Sample sizes: Malawi (n = 101), Ghana (n = 183), Cambodia (n = 270), and PNG (n = 72).

While the crystal structure of EBA-175-RII has been solved [53], we used a 3D7-based *ModPipe* homology structure for analysis, as this model includes a number of functionally important residues that were not resolved in the experimental structure [16]. For D* analysis on EBA-175-RII, a large region of the F1 domain which predominantly consisted of AA residues E226—L294, I312 –K324, and W377 –I400 was under balancing selection (i.e. high D* scores of 2.8–3.0) in all parasite populations. This cysteine-rich region is also involved in the dimerization interface formation (helix linker and disulphide bridges)[53] between two molecules of EBA-175-RII as it binds to human receptor glycophorin A. During dimerization, this region also makes contact with another cysteine rich F2 domain of the other dimer pair in a ‘handshake’ interaction [53]. However, slight variations between African and Asia-Pacific populations can also be found within the site from F2 domains, which are predominantly comprised of AA residues C476–C488 and Y710 –F722 where high D* scores (1.8–2.0) were observed within most Asia-Pacific countries, but not African countries (D* scores < 0.4) (Fig 6A). The F2 domain makes most of the contact with glycan [54] and residues C476–C488 are a part of F2 β-finger domain that is also a target of inhibitory antibodies, R215 and R217 [54,55]. The linker region was found to be conserved within all populations (Fig 6A). However, the limited nucleotide diversity was observed amongst geographical regions.

The cryo-EM structure of full length RH5 (PDB Code: 6MPV, chain B) was used in our analysis [56]. Evidence of balancing selection was limited within African countries parasite populations based on the spatially derived Tajima’s D* scores. In contrast, within Asia-Pacific countries, moderate to high D* scores (1.5–2.0) were consistently observed at the Basigin (BSG or CD147) binding sites and appear to be under balancing selection (AA residues: S189 –D207, 3D7 sequence) (Fig 6B). Additionally, the residues near CyRPA binding sites which predominantly consist of residues I386 –F421 have moderate evidence of balancing selection (D* score of 1.5) within PNG population (Fig 6B). This suggests the BSG binding site may be a key target of host immunity and aligns with previous findings from Alanine *et al*. (2019) [57]. Non-synonymous AA polymorphisms at residues Y147 and H148 (nucleotide positions 439, and 442, 3D7 sequence) were under balancing selection in most Asia populations but appear to be under adaptative selection in some African populations according to the linear sliding window analysis with *Tajima*’s *D* values around 1.7 (S3 Fig). These residues did not have PDB coordinates (physical proximity to N-terminal disordered region), and therefore cannot be detected via spatially derived 3D analyses. Nucleotide diversity of RH5 was slightly higher in Asia-Pacific populations than African populations (S3 Fig).

We observed limited evidence of balancing selection (near neutral) within the thrombospondin receptor domain of CSP [58,59] for Asia-Pacific populations (Cambodia and PNG). However, moderately high D* scores (1.2–1.3) were observed at Th2R residues (AA residues E310 –L327, 3D7 sequence) at Malawi and Ghana populations which suggests some evidence of balancing selection (Fig 6C). Similarly, nucleotide diversity of some of the residues comprising thrombospondin receptor domain of CSP were relatively low in Asia-Pacific populations compared to African populations (S7 Fig). A moderately high to high degree of balancing selection (D* scores of 1.0 and 2.9) for Th3R residues (AA residues G341 –I364) was also observed within African populations (Fig 6C).

Moderately high D* scores (1.0–2.0) are found within residues Q1612 –F1625, and E1632 –C1647 from African populations (Malawi and Ghana), but not in Asia-Pacific populations (Fig 6D). Most of these residues were a part of conformational epitopes and involved in interaction with potent monoclonal antibody (G17.12) [60] and inter-domain interactions. In addition, nucleotide diversity of these residues is relatively high in African populations compared to Asia-Pacific parasite populations (S8 Fig). However, different part of MSP1-19 with residues P1651 –C1682 were under balancing selection in most of the Asia-Pacific populations (only shown here for Cambodia and PNG populations) (Fig 6D).

Hotspots of balancing selection were found within N-terminal AA residues F87 and D93 –S104 (3D7 sequence) of CelTOS [61] for most of the populations with moderately high to high spatially derived Tajima’s D* scores (1.0–2.2) (S7A Fig). Within the Malawi population, we found high D* scores (1.8–2.0) at AA resides D131 –I138 (3D7 sequence), but limited balancing selection was found for CelTOS within PNG (D* scores < 0.4) (S7A Fig). Nucleotide diversity was also relatively low in PNG populations compared to others (S7B Fig). However, the presence and distribution of D* values were variable amongst geographic locations (S7A Fig). Limited functional information was available for CelTOS therefore the significance of these findings is not able to be postulated.

### Intrinsically disordered proteins may be targets of immune selection

**Fig 7:**
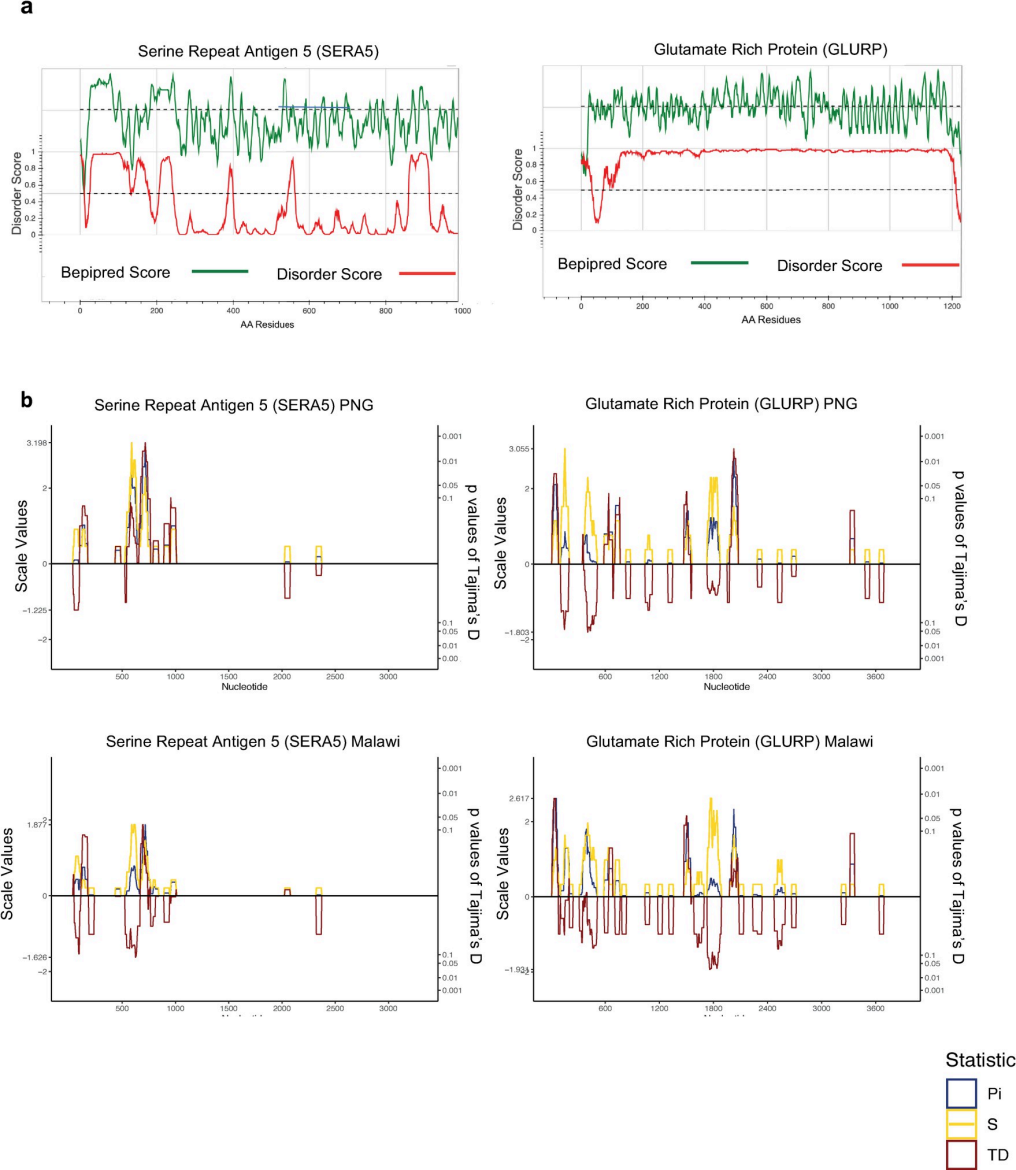
Diversity and selection of SERA5 and GLURP in Asia-Pacific and African regions. a) Computational predictions of protein disorder and B-cell epitopes in SERA5 and GLURP. The green line represents the linear B-cell epitope mapping scores and the red line shows the protein disorder score, respectively. b) Diversity statistics along the sera5 and glurp genes in samples from in Asia-Pacific and African regions, represented by Tajima’s D (red line), nucleotide diversity (blue line) and number of segregating sites (yellow line). It is calculated in the context of linear sequence level based on coding region with the sliding window approach (a window size of 50 bp and a step size of 5 bp). Nucleotide positions based on coding region are shown in the x-axis. Sample sizes: Malawi (n = 106), Ghana (n = 208), Cambodia (n = 405), and PNG (n = 108).

Intrinsically disordered proteins (IDP) and intrinsically disordered regions (IDR) are a class of proteins, which lack secondary and tertiary rigid structure (under physiological conditions) but possess active roles in many biological processes [62–71]. Recent predictions have shown an abundance of IDPs within the *Plasmodium* proteome including several vaccine candidate antigens [28]. This is not surprising given that disordered regions create larger intermolecular interfaces, which increases the chances of interaction with potential binding partners, even without tight binding, providing flexibility for binding diverse ligands and other proteins including functional antibodies [29]. IDPs can have a diverse range of functions, although our understanding of the role of IDPs within biological systems is still incomplete [64,68]. Due to the lack of well-defined three-dimensional structure for antigens with extensive disordered regions, we calculated linear *Tajima’s D* values to determine balancing selection. Highly disordered proteins were defined as those with more than 50% residues with disorder scores higher than 0.4. This includes several emerging and established vaccine candidate antigens such as CTRP, EXP1, TRAP (C-terminal), STARP, GLURP, EBA175 (RIII-V), MSP3, MSP4, MSP6, RALP1, SERA5 and RESA. For these antigens except STARP and RALP1, evidence of balancing selection was consistently observed within disordered regions with only slight variations amongst geographical regions (Figs 7 and S10). Intrinsically disordered regions (IDR) were previously shown to be enriched with predicted linear B-cell epitopes [28]. We found disordered residues within these antigens (i.e. N-term of SERA5 compared to its C-term [72,73]) contributing to balancing selection (*Tajima’s D* values >1) at most of the analysed geographic area or countries (Fig 7). This suggests IDRs as potential immune targets. However, it has been estimated that IDRs have limited proportion of MHC-binding peptide compared to other domains, which may affect T-cell dependent immune responses [28]. Further *in vitro* and *in vivo* investigations are needed to characterize antigens enriched with disordered proteins as potential vaccine targets.

## Discussion

Here we have conducted a systematic meta-population genetic analysis of 23 malaria vaccine antigens using a global dataset of *P*. *falciparum* WGS (after filtration for high complexity infections) covering 15 malaria-endemic countries [22]. Our results demonstrate low frequencies of variants included in malaria vaccines under development, and variable levels of diversity and natural selection amongst different antigens and also amongst different geographic regions. Overall, the results demonstrate that the current ‘one size fits all’ approach to vaccine development may result in limited vaccine efficacy and variable efficacy across geographic regions [74]. Different approaches to vaccine design may include focusing on the most common haplotypes circulating in the population, and not limiting to the reference strain by for example, basing the vaccine on the most common haplotypes as shown in Table B in S1 Text.

The variable success of malaria vaccines may be due to the high diversity of vaccine candidate antigens [7,15,75] and strain-specific immune responses. Merozoite antigens, particularly AMA1, and EBA175 have high haplotype and nucleotide diversity across different populations, consistent with strong immune selection. Variable levels of nucleotide diversity for different populations were observed for CSP (C-term) and CelTOS which may result from variable transmission intensity in different regions. Low nucleotide diversity and moderate haplotype diversity across populations for STARP, RH5, and RALP1 suggests that although there may be low polymorphism, immune selection maximises the chance of new infections carrying novel haplotypes. Only limited haplotype and nucleotide diversity was observed within CyRPA, TRAMP, and Pfs48/45 suggesting strong conservation and limited immune selection. For STARP, RH5, RALP1, and CyRPA antigens, low expression throughout the erythrocytic stage (based on abundance of *in vivo* mRNA transcript) suggests minimal opportunities for host evolutionary pressure to be exerted upon those antigens [68,76].

Previous studies have indicated that parasite antigens are differentially immunogenic [77] and this manifests in varying intensity of immune (balancing) selection. We have detected extremely high balancing selection across different populations for AMA1 and EBA175 (RII), moderate to high balancing selection was found for TRAP (ectodomain) and RIPR. Whereas CyRPA, and SERA5 (C-term) appear to have consistently low diversity, and are neutrally evolving in most populations, which implies they have highly conserved epitopes or weak immunogenicity. Variable selection amongst different populations was found for RH5, CSP (C-term), CelTOS, MSP-19 and Pfs48/45 (S9 Fig). High-affinity, receptor-specific binding between parasite ligand and host receptor (such as EBA175 –Glycophorin A) or low-affinity general cell binding (such as TRAP–heparan sulphate proteoglycans (HSPGs)) may explain different balancing selection intensity on these antigens amongst populations [42,55,78]. The high relative solvent accessibility (RSA) of polymorphic residues on some of these antigens suggests they may be antibody targets, supported by considerable amino acid variability, as measured by Shannon Entropy, at host-parasite interfaces, particularly for the TRAP ectodomain, CSP C-term, AMA1, EBA175 RII, RH5, Pfs48/45 6C domain, and CelTOS. All of these antigens have selection hotspots at these interfaces in at least one population.

Hotspots of balancing selection were common within functionally important 3D interfaces of AMA1 and TRAP across populations suggesting sharing of dominant epitopes which may allow the development of broadly efficacious vaccines using a few common variants. On the other hand, we identified differential patterns of selection pressure between African and Asia-Pacific countries at the ligand binding sites of EBA175 RII, RH5, MSP1-19 and CSP C-terminal end. Even more discordant patterns between geographic regions were found for gametocyte antigens such as CelTOS and Pfs48/45. This might reflect parasite adaptation to different host environments. These results were aligned with haplotype network analysis. For blood stage antigens, EBA175 and RH5, this could be driven by different isoforms of the respective host receptors such as glycophorin-A (GPA) [79] and Basigin [80]. GPA encoded by the *GYPA* gene is under selective pressure in different malaria endemic areas, with the MN blood group in particular showing protective associations against malaria in PNG [79,81]. Associations between different isoforms of BSG with malaria outcomes have not yet been identified however *in vitro* studies have shown a reduced RH5 interaction with isoform 2 of BSG [80]. Despite the very low diversity of RH5 there are multiple common haplotypes, with strong balancing selection at the BSG binding site. However, this balancing selection was only found within Asia-Pacific populations. This suggests that RH5 may have adapted to an unidentified BSG isoform in the region, or that some other factor might be contributing to selection at this BSG-binding interface. Similarly, CSP C-terminal and CelTOS shows unique selection hotpots on the 3D structure as these proteins are predominantly expressed in the sporozoites, this might reflect adaptation to different Anophelene species in Africa and Asia. This geographically variable selection was not easily observed with sliding window analysis of linear sequences showing the enhanced resolution of the 3D analysis.

Immune responses may also be generated to conserved regions of antigens [82] such as that in the novel universal influenza B-cell vaccine approach [9]. This would increase vaccine efficacy for diverse strains by eliciting broadly cross-reactive functional immune responses such as neutralising antibodies directed at highly conserved regions [83–86]. Conserved regions of malaria vaccine candidate antigens have been revealed within RH5, CyRPA, or SERA5 C-terminal end (S8 Fig) and a lack of selection pressure in the C-terminal region of SERA5 and CyRPA. Therefore, not only does this study catalogue and characterise global malaria vaccine antigen diversity, it can also guide the identification of conserved regions. This study therefore provides critical practical information for the next generation of malaria vaccines.

The immunogenic properties of disordered proteins are not well understood. Lower immunogenicity of a disordered region compared to a structured region within MSP2 has been observed [87] suggesting that disordered proteins fail to activate the production of high affinity, specific antibodies, because they adopt diverse conformations depending on their ligands [88]. Therefore, polymorphic residues on disordered proteins may be a ‘smoke screen’ by diverting protective immune responses from more ordered targets. Nonetheless, numerous B-cell epitopes have been found within disordered regions [28] and many of these appear to elicit functional immune responses [70,71,75,89–92]. For instance, the protective effect of RTS,S appears to be predominantly mediated by generation of antibody responses to the disordered central NANP repeat region [90,93]. Our observation of immune-mediated selection pressure in the N-terminal disordered region of SERA5, is also supported by the finding that *in vitro* parasite growth was inhibited by anti-SERA5 antibodies raised in squirrel monkeys (*Saimiri sciureus*) [72]. In addition, we found balancing selection in the disordered EBA175 RIII-V domain which may facilitate the molecular binding of GYPA to EBA175 RII [94,95]. Future work could extend to characterise disordered proteins into different subclasses based on their immunogenicity.

This study presents a detailed analysis of the genetic diversity and immune selection of 23 candidate vaccine antigens and a framework for analysis of further antigens which has already contributed critical information about the diversity of novel vaccine candidates [96–98]. In addition, the incorporation of sequence-based metrics such as Tajima’s *D* into protein structures enhances biological understanding, as evidenced by the finding of unique patterns of immune selection in different geographic areas. However, the dataset mostly includes single census of clinical samples from different collection timepoints, which prevent the exploration of patterns under different clinical presentations and changes in transmission. Further investigations could be done using sequencing datasets from longitudinal cohorts in association with epidemiological parameters to identify specific polymorphisms that enable the parasite to evade host immune responses. Nevertheless, *in silico* findings from our study support *in vitro* and *in vivo* studies and might uncover novel targets of host immunity. The development of a novel population genetic analysis software ‘VaxPack’ was crucial to automate the data analysis. The approach used in this work is also applicable to a wide variety of problems from different organisms.

## Materials and methods

### Data sources

WGS data from *P*. *falciparum* clinical isolates (n = 2512) from multiple countries including Bangladesh, Cambodia, Laos, Myanmar, Thailand, Vietnam, Congo (DRC), Malawi, Gambia, Ghana, Guinea, Mali, Nigeria and Senegal were obtained from the MalariaGEN Pf3k project release 5.1 as unmapped BAM (uBAM) files [10]. WGS data from *P*. *falciparum* isolates from PNG (n = 156) were also obtained from the MalariaGEN *P*. *falciparum* Community Project [23] giving a total of 2668 WGS for data analysis. All samples were sequenced using Illumina short read technology at the Sanger Research Institute, UK, and the Broad Institute, Boston, USA [10,24]. The sequences from these 15 countries cover four geographical regions (Southeast Asia, Central Africa, West Africa, and Oceania) with variable malaria transmission intensities [99].

### Processing and analysis of whole genome sequence data

*P*. *falciparum* read alignments from all 2668 samples were mapped to an indexed 3D7 reference genome using uBAM files as input. We followed the standard best practice from *Genome Analysis Toolkit (GATK)* version 4.0.12.0 implemented in *nextflow* (https://github.com/gatk-workflows/gatk4-germline-snps-indels) with some minor changes as follows: (i) running the pipeline twice—the first without base quality score recalibration (BQSR) and (ii) using the pass variants from the first run for BQSR and hard filtering of variants instead of variant quality score recalibration (VQSR) as there is no external variant call set available. Variants were determined using GATK’s *haplotypeCaller* to generate haploid genotype calls (*P falciparum* blood stages are haploid) and all isolates were joint genotyped using gvcf files. Variants that had been processed with the GATK pipeline were functionally annotated with *SnpEff for* genomic variant annotations and functional effect prediction (version 4.3T) [100]. We restricted our analyses to single nucleotide polymorphisms (SNPs) from the 14 nuclear chromosomes only, excluding indels and variants from hypervariable regions, as they evolve by different mechanisms to SNPs and have much less impact on antigen genes (S1 Fig). To obtain a high-quality variant dataset for downstream analyses, we further developed stringent variant filters using GATK’s SelectVariants and VariantFiltration modules such as read coverage statistics, relative distribution of reads [101] and multiplicity of infection (MOI) (S1 Fig). Variants were removed if they met at least one of the following filtering criteria: QD < 20.0, MQ < 50.0, MQRankSum < -2 (if annotated), ReadPosRankSum (less than -4 or greater than 4 if annotated), SOR > 1. Further filtration was performed based on read depth, and missingness (S1 Fig). We estimated the clonal proportions for each sample using R package, *moimix* (available at https://github.com/bahlolab/moimix), a robust alternative (B)-allele frequency-based estimation. We only kept data from samples with single infections (MOI = 1) or two infections (MOI = 2; i.e. Fws > 0.80) [24]. MOI = 2 isolates were treated as diploid genomes permitting heterozygous genotype calls (S2 Fig). Multi-allelic sites were also permitted in our bioinformatics framework (S1 Fig). All samples that had F_ws_ greater than 0.90 were treated as MOI = 1. For MOI = 2 samples, phasing of haplotype was performed using read-depth and B-allele frequency (S1 Fig). Thus, the final dataset contained high quality data from 948 MOI = 1 and 551 MOI = 2 isolates. Nucleotides that did not have clear major or minor alleles were classified as ‘undefined’ bases (S1 Fig). Variants within gene coding regions were extracted in fastA format using a custom R-script (https://github.com/myonaung/Naung-et-al-2021). After processing the sequence data, identifying high quality variants and removing complex infections, gene sequences were extracted and compiled as a fastA formatted file.

### Processing of antigen sequences

Antigens were selected based on their inclusion as vaccine candidates in the WHO Rainbow Table [20] or whether they had been previously identified as novel candidates based on previous literature [6]. Details of regions analysed, and other details are summarised in Table 1. The raw fastA output for each gene was further edited using customized python script (https://github.com/myonaung/Naung-et-al-2021) by removing sequences that contain undefined or low-quality bases and sequences arising from minor clones in the co-existence of different genotypes (phased by read depth (S1 Fig)). FastA formatted files of respective genes can be found in https://github.com/myonaung/Naung-et-al-2021/tree/master/Raw_FASTA. As Illumina sequencing is subject to some miscalled bases, singleton variants (i.e. minor alleles seen only once in the entire dataset) were assumed to be artefacts and converted back to reference alleles to prevent false positive variants.

The presence of at least two isolates with the same minor allele was considered independent validation of the existence of these alleles in natural parasite populations. Apart from removing singletons, no other minor allele frequency threshold was applied to the dataset. In the case of multiple clones being identified (0.90 ≥ Fws > 0.80), only the defined major alleles were included for further analyses. Samples collected from the same country were combined and assumed to have been sampled from a single population.

### Population genetic analyses

Population genetic parameters were calculated using a customized in-house R package (source code available at https://github.com/BarryLab01/vaxpack). This program takes aligned multi-fastA files excluding intronic regions as input, alongside reference sequence of the same length. As measure of diversity we defined the polymorphisms for each antigen by the number of polymorphic sites (S), the number of synonymous (dS) SNPs, number of nonsynonymous (dN) SNPs, dN-based haplotypes, the Nei’s nucleotide diversity (π) calculated as

$$\pi =\sum_{ij}x_{i}x_{j}\pi_{ij}$$ (1)

where *x_i_* and *x_j_* are the respective frequencies of the i^th^ and j^th^ sequences, *π_ij_* is the number of nucleotide differences per nucleotide site between i^th^ and j^th^ sequences [92]. The haplotype diversity (Hd) was defined as

$$Hd=[n/(n-1)][\left(1-\Sigma {\left(f_{i}\right)}^{2}\right)]$$ (2)

where n is the sample size and f is the frequency of the i^th^ allele. Immune mediated selection due to parasite adaptations to evade antibody recognition of dominant epitopes on a particular antigen is indicated by the presence of balancing selection. To measure selection, *Tajima’s D* (*D*) statistics were calculated [102,103]. Under neutral conditions, *Tajima’s D* values are expected to be approximately zero [104,105], positive *D* values indicate an excess of intermediate frequency polymorphisms most likely due to balancing (immune) selection, and negative values indicate purifying (directional) selection. However, measurement of *Tajima’s D* across genetically differentiated populations with varying allele frequencies (i.e. due to population structure) can produce false positive signals of balancing selection. Thus, all *Tajima’s D* analyses were calculated separately for each country. Analyses were first carried out with the complete global dataset (n = 1499 samples) for all antigens from Table 1 to investigate the global range of diversity of each antigen as well as the frequency of reference (3D7) haplotypes (frequently used in vaccine development), while the population specific dataset was used to investigate the range and distribution of diversity for countries (23 antigens). RON2 (PF3D7_1452000) was excluded from analyses as no polymorphism was found. The full-length antigen genes as well as functionally important domains were included in the country level.

For each analysed antigen gene, we investigated relationships amongst haplotypes by constructing haplotype networks using the Templeton, Crandall, and Sing (TCS) method in *PopArt* version 1.7 [106]. To focus the analysis on antigen diversity, we based our analysis on non-synonymous polymorphism (via amino acid translation) as synonymous polymorphisms do not change the protein structure. It is very important to note that haplotypes are simply the combination of polymorphic sites without a particular weight upon a specific or functionally important polymorphic residue. All nonsynonymous SNP haplotypes with a frequency greater than 0.5% of total population were included in the analysis using ‘Nexus’ file format as input. The TCS network was constructed using an agglomerative approach. This allows for visualization of the relationships amongst haplotypes and their geographic distribution. Generally, less frequent recombinant haplotypes connect major (high frequency) haplotypes.

The Relative Solvent Accessibility (RSA) derived from the solvent accessible surface area (ASA) was predicted for all antigens for each amino acid residue based on primary 3D7 protein sequence using neural network based NetSurfP1.1 program [30,107]. It is given by the following equation [30,107]:

$$RSA=ASA/ASA_{MAX(Gly‐X‐Ala)}\;*\;100\%$$ (3)

RSA values for structured regions with known PDB or homology-modelled structures were calculated with DSSP [31] using maximum allowed solvent accessibilities of residues from Tien *et al*. 2013 [107]. Therefore, RSA represents the ratio of parts of a biomolecule exposed to solvent or accessible surface area (ASA) of a given residue observed in the three-dimensional state over maximum surface area of a residue with potential exposure to solvent (ASA_MAX_) within an extended tripeptide flanked with either glycine or alanine residue. The data was stored in SQLite database using RSQlite package on RStudio.

Selection pressure may apply to residues that are non-continuous on the linear sequence but proximal in 3D space. Therefore, application of a more accurate spatially derived approach to compute Tajima’s D* and nucleotide diversity is applicable. We chose the radius of 15 Å to reflect the ideal potential antibody antigen interaction as used by Guy *et al*. (2018) [16,17]. However, our spatial averaging approach was limited to the availability of 3D coordinates for each amino acid residue, and therefore were not applied to highly disordered regions or proteins.

### 3D structure-based and surface accessibility analysis

For each antigen included in Table 1, the 3D structure for full length or specific domains was first derived from the Protein Data Bank (PDB) from the Research Collaboratory for Structural Bioinformatics (RCSB) website (www.rcsb.org). For each antigen that does not have experimentally determined or complete structure, the possibility of comparative structure modelling was determined based on the distribution of intrinsic disordered regions available at https://plasmosip.burnet.edu.au/submission [28]. Except for AMA1, which was modelled manually, template-based models of 3D7 reference strain for these antigens were predicted using *ModPipe*, an automated software pipeline that utilises the program *MODELLER* [108] for the generation of comparative proteins based on previous studies [16]. We used a manually modelled structure of AMA1, which includes full domains I-III based on a combination of *P*. *falciparum* and *P*. *vivax* templates according to previously published work [27]. The modelled structures were assumed to be reliable if the *ModPipe* Quality Score (MPQS) is greater than 1.1 This included for antigens–EBA175 (RII), MSP1_19_, and CelTOS which is 45% sequence identity to *P*. *vivax* CelTOS structure (PDB Code: 5TSZ) [61]. These structure models are accessible via *ModBase* (https://modbase.compbio.ucsf.edu/) [108]. The *BiostructMap* Python package available at https://github.com/andrewguy/biostructmap was used to calculate nucleotide diversity (π), and *Tajima’s D* with consideration of spatial information using a 3D sliding window with a radius of 15 Å (D* = spatially derived D). Spatial *Tajima’s D* (D*) with 3D sliding window is analogous to traditional sliding window analysis that should not be interpreted at individual residues since it averages out contributions from individual residues. Since the spatial *Tajima’s D* (D*) calculation also considers the spatial information of each amino acid residue from the protein, the results represent accurate detection of selection pressures arising at the level of protein structure but might be variant from the traditional linear *D* calculation as shown in Guy *et al*. (2018) [16,17]. D* scores of 1.0 to 1.9 were considered as moderately high and D* scores of above 2 are high. Protein structures and features were visualised using *PyMol* with feature values saved into the B-factor column of a PDB file and using the *spectrum* command. We estimated disordered scores using the PlasmoSIP online tool (https://plasmosip.burnet.edu.au), which contains precalculated results from DISOPRED3 [66]. Due to the lack of well-defined three-dimensional structure for antigens with extensive disordered regions, we calculated linear *Tajima’s D* values to determine balancing selection.

Normalized Shannon Entropy for each amino acid residue mapped onto the available protein structure was calculated using

$$S=\sum_{i}\frac{p_{i}{log}_{2}p_{i}}{{log}_{2}\;20}$$ (4)

where S is the normalized Shannon Entropy, and p_i_ is the frequency of i^th^ amino from the specific position [90]. The normalized Shannon Entropy is a site-specific diversity measure ranging between 0 to 1 where 0 represents the conservation of a specific amino acid position, and 1 represents an even distribution of all possible standard 20 amino acids (random distribution) at the specific position. The analysis was limited into antigens with available or predicted 3D protein structure.

## Supporting information

S1 Fig Variant Processing Framework. A pre-processing phase specifies GATK’s Haplotype caller’s SNPs variants from the regions of interest and performs quality control on these variants. The hard-filtering phase selects only high-quality variants from the variants that passed the pre-processing phase. The integrative phase removes polyclonal (> MOI 2), performs haplotype phasing, and extracts sequences for gene of interest.
(TIFF)

S2 Fig F_ws_ output from moimix R-package for each country. F_ws_ > 0.90 assumed as MOI 1 isolates are highlighted in red, and 0.90 ≥ Fws > 0.80 assumed as MOI 2 isolates are highlighted in blue. Samples with F_ws_ below 0.80 are excluded from the analysis.
(TIFF)

S3 Fig Polymorphism and selection of full-length rh5 in the context linear sequence level for different populations. The sliding window analyses (a window size of 50 bp and a step size of 5 bp) calculated for segregation sites (S, yellow lines), nucleotide diversity (π, blue lines) and Tajima’s D (D, red lines) for each geographic area or country. The results were plotted together and scaled to Tajima’s D values. Nucleotide positions based on coding region are shown in the x-axis. The significant values for Tajima’s D was determined based on sample size.
(TIFF)

S4 Fig Geographically varied selection pressure for SERA5. Tajima’s D (D*) calculation for geographic area or countries from Asia-Pacific and African regions for SERA5 (C-terminal) with incorporation of protein structural information using 15°A window. The structured region of SERA5 based on experimentally defined structure PDB code: 2WBF was used. The structure was coloured according to D* scores mapped to each residue with undefined D* were shown in grey. Only Malawi (n = 106), and PNG (n = 108) populations were shown.
(TIFF)

S5 Fig Geographically conserved spatially derived nucleotide diversity for full-length AMA1. Nei’s nucleotide diversity calculation for geographic area or countries from Asia-Pacific and African regions for AMA1 with incorporation of protein structural information using 15°A window. Structure was coloured according to nucleotide diversity mapped to each residue. Sample size for each respective population are as follows: Malawi (n = 139), Ghana (n = 243), Cambodia (n = 433), and PNG (n = 112). Similar to selection pressure (determined by D*), silent face of AMA1 has low nucleotide diversity.
(TIFF)

S6 Fig Geographically variable spatially derived nucleotide diversity for CSP (C-term). Nei’s nucleotide diversity calculation for geographic area or countries from Asia-Pacific and African regions for CSP (C-term) with incorporation of protein structural information using 15°A window. Structure was coloured according to nucleotide diversity mapped to each residue. Sample size for each respective population are as follows: Malawi (n = 135), Ghana (n = 223), Cambodia (n = 431), and PNG (n = 111).
(TIFF)

S7 Fig Geographically variable selection for CelTOS. A. Tajima’s D (D*) calculations for populations from Asia-Pacific and African regions for CelTOS with incorporation of protein structural information using 15 Å window. Structure was coloured according to D* scores mapped to each residue with undefined D* were shown in white. 3D7-based ModPipe model of the P. vivax CelTOS based on 5TSZ template was used. Sample sizes: Malawi (n = 142), Ghana (n = 245), Cambodia (n = 433), and PNG (n = 112). B. Nei’s nucleotide diversity calculation for geographic area or countries from Asia-Pacific and African regions for CelTOS with incorporation of protein structural information using 15°A window. Structure was coloured according to nucleotide diversity mapped to each residue. Sample size for each respective population are as follows: Malawi (n = 142), Ghana (n = 245), Cambodia (n = 433), and PNG (n = 112).
(TIFF)

S8 Fig Geographically variable spatially derived nucleotide diversity for MSP1-19. Nei’s nucleotide diversity calculation for geographic area or countries from Asia-Pacific and African regions for MSP1-19 with incorporation of protein structural information using 15°A window. Structure was coloured according to nucleotide diversity mapped to each residue. Sample size for each respective population are as follows: Malawi (n = 101), Ghana (n = 183), Cambodia (n = 270), and PNG(n = 72).
(TIFF)

S9 Fig Geographically varied selection pressure for Pfs48/45. The sliding window analyses (a window size of 50 bp and a step size of 5 bp) calculated for Tajima’s D (D, red lines) for each population. Nucleotide positions based on coding region are shown in the x-axis. Significant value for Tajima’s D was determined by sample size. Sample size for each respective population are as follows: Malawi (n = 142), Ghana (n = 247), Cambodia (n = 433), and PNG (n = 112).
(TIFF)

S10 Fig Selection of disordered proteins in Asia-Pacific and African regions. a) Computational predictions of protein disorder and B-cell epitopes in EBA175, MSP3, MSP4, MSP6, RESA, TRAP, EXP1 and CTRP. The green line represents the linear B-cell epitope mapping scores and the red line shows the protein disorder score, respectively. b) Tajima’s D statistics along the disordered antigens in samples from Cambodia, PNG, Malawi, and Ghana. It is calculated in the context of linear sequence level based on coding region with the sliding window approach (a window size of 50 bp and a step size of 5 bp). Nucleotide positions based on coding region are shown in the x-axis. Sample size for each respective population are as follows: Malawi (n = 106), Ghana (n = 208), Cambodia (n = 405), and PNG (n = 108).
(TIFF)

S11 Fig Nucleotide sequence variability of all antigens included in the study based on all available sequences. Sliding window analysis of sequence variability was calculated using algorithm from Proutski and Holmes *et al*., (1997) [1] implemented in MBEToolbox [2] using default parameters on MATLAB (version R2020a). Mean (red line), and standard deviation (green dotted line) within each antigen are shown. Nucleotide positions based on coding region are shown in the x-axis.
(TIFF)

S1 Text Supporting Materials. **Table A:** Antigen Diversity Summary (Full length or specific domain). **Table B:** Prevalence of haplotypes in dataset for analysed antigens and their proximity to 3D7 vaccine haplotype.
(DOCX)
