# Supplementary figures and images for: Global diversity and balancing selection of 23 leading Plasmodium falciparum candidate vaccine antigens
Source: PLoS Comput Biol. 2022 Feb 2;18(2):e1009801. doi: 10.1371/journal.pcbi.1009801 (PMC8843232; doi:10.1371/journal.pcbi.1009801)

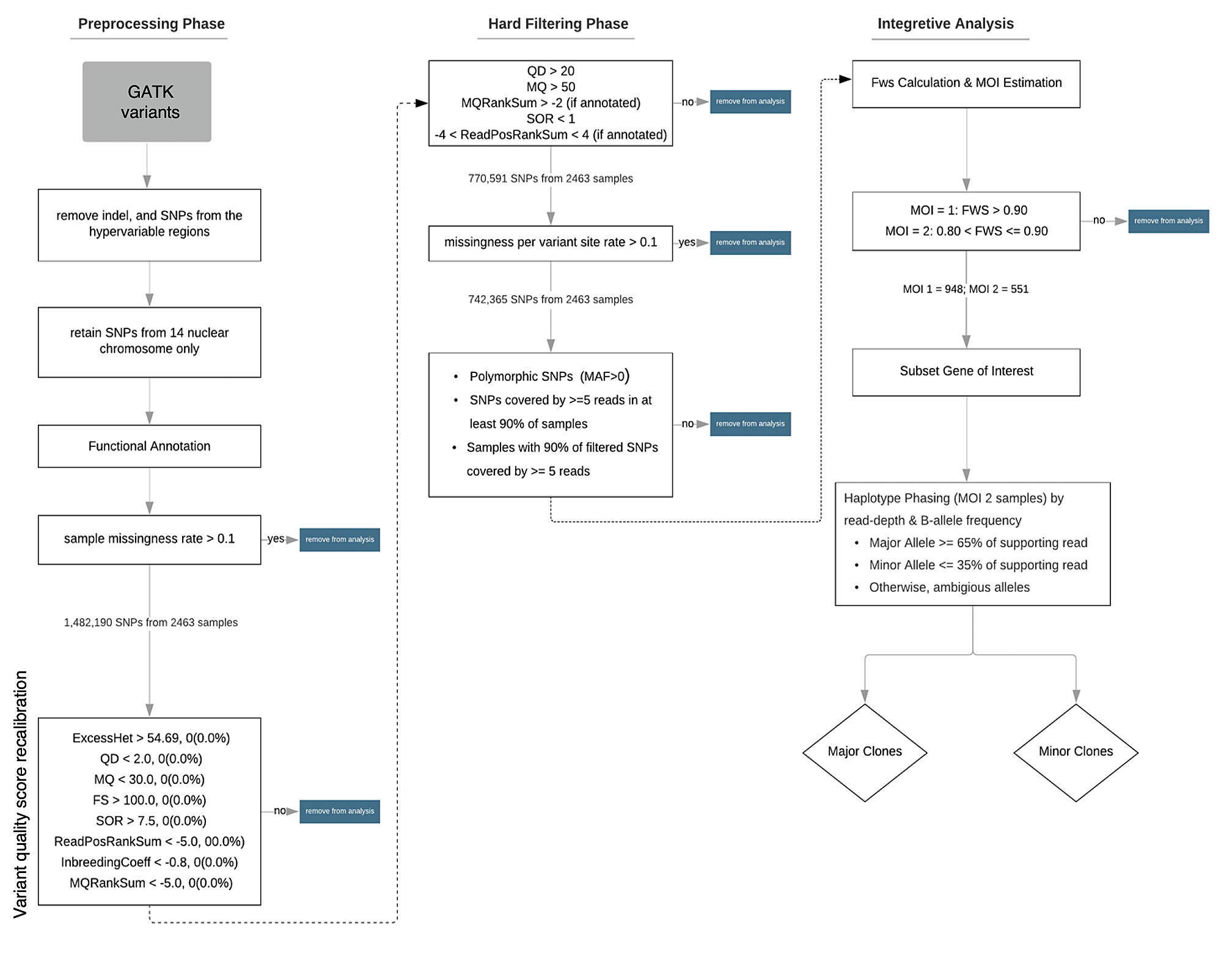

Supplement: S1 Fig — A pre-processing phase specifies GATK’s Haplotype caller’s SNPs variants from the regions of interest and performs quality control on these variants. The hard-filtering phase selects only high-quality variants from the variants that passed the pre-processing phase. The integrative phase removes polyclonal (> MOI 2), performs haplotype phasing, and extracts sequences for gene of interest. (TIFF) [file pcbi.1009801.s001.tiff]

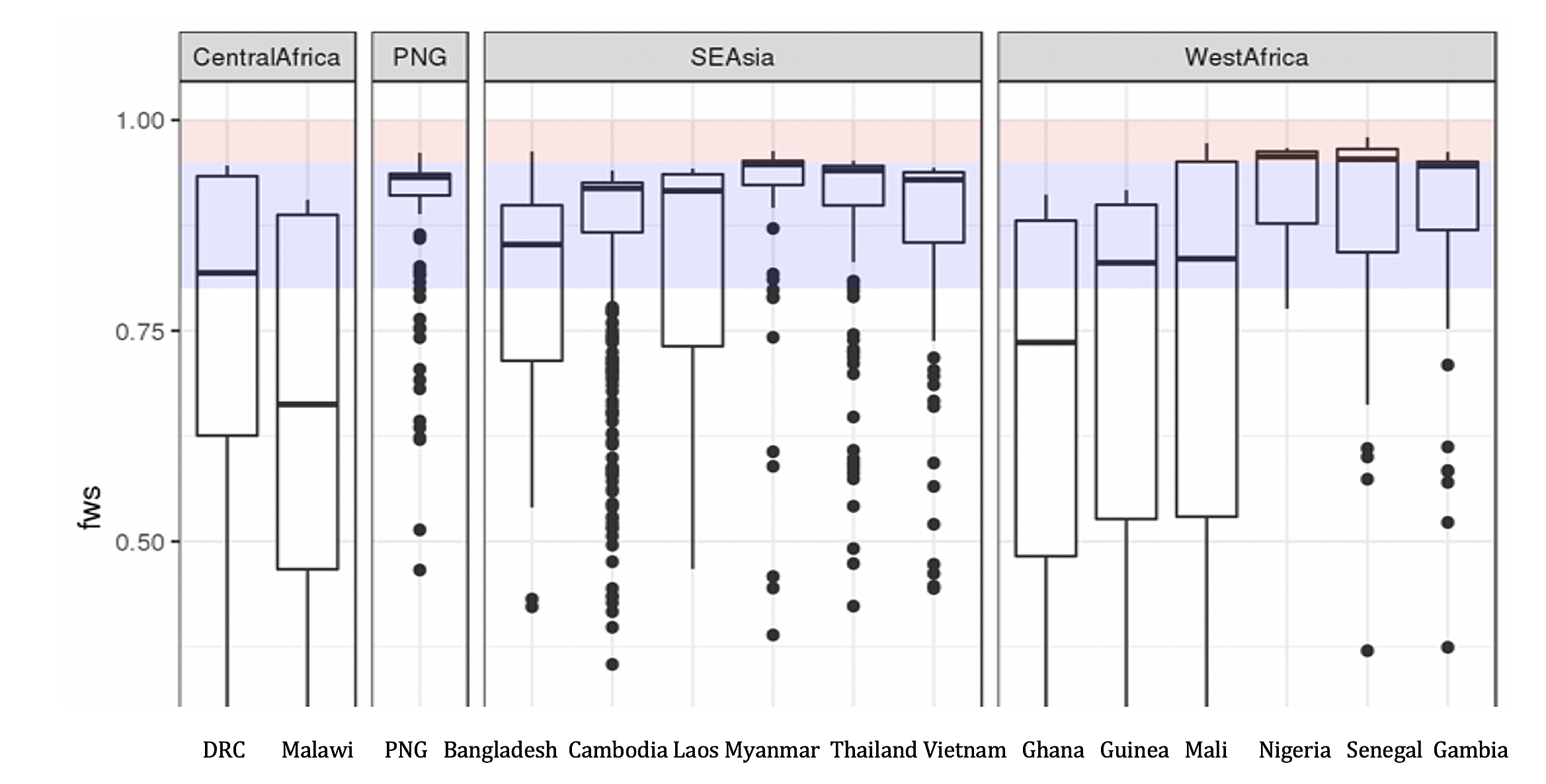

Supplement: S2 Fig — Fws > 0.90 assumed as MOI 1 isolates are highlighted in red, and 0.90 ≥ Fws > 0.80 assumed as MOI 2 isolates are highlighted in blue. Samples with Fws below 0.80 are excluded from the analysis. (TIFF) [file pcbi.1009801.s002.tiff]

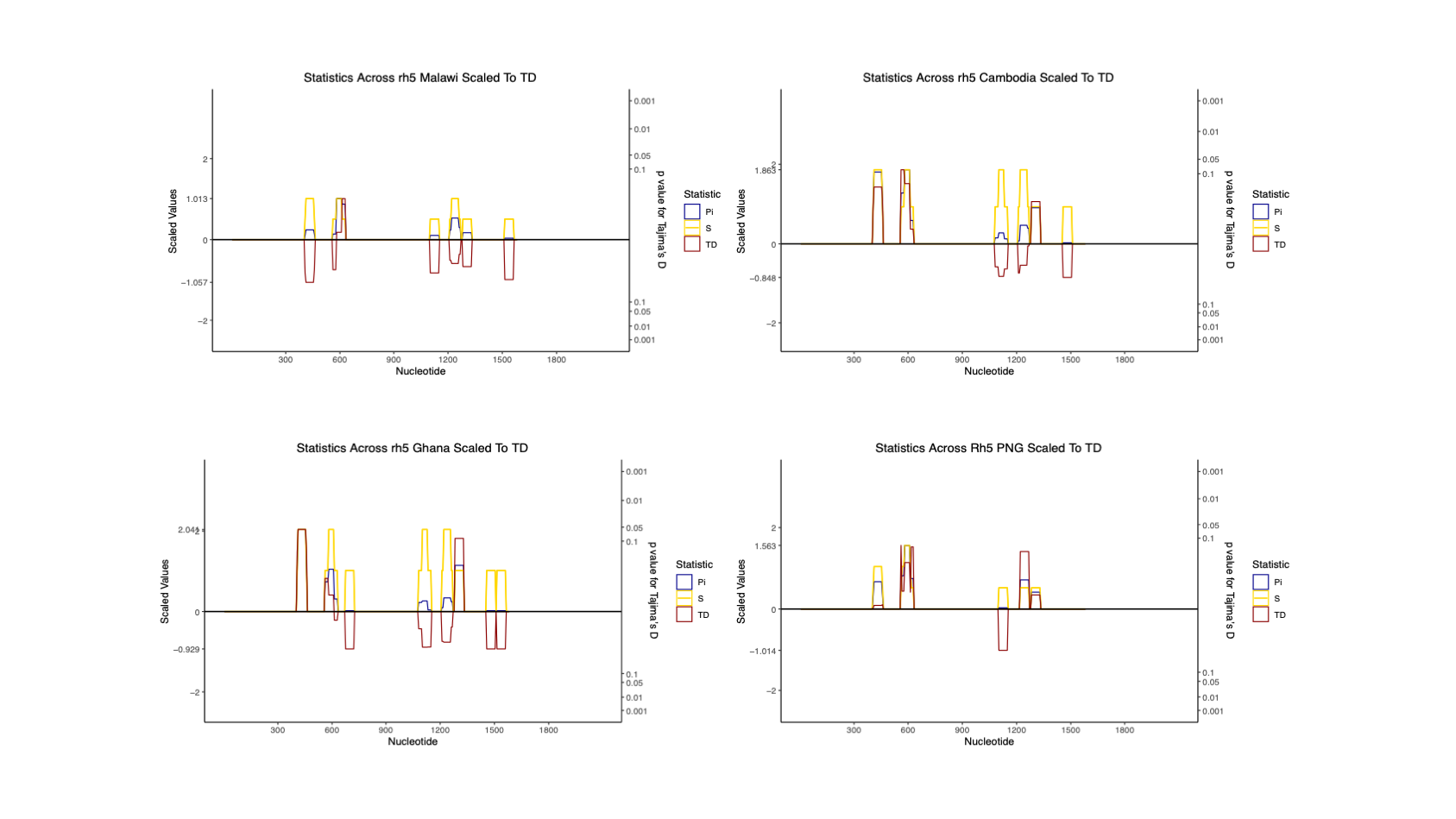

Supplement: S3 Fig — The sliding window analyses (a window size of 50 bp and a step size of 5 bp) calculated for segregation sites (S, yellow lines), nucleotide diversity (π, blue lines) and Tajima’s D (D, red lines) for each geographic area or country. The results were plotted together and scaled to Tajima’s D values. Nucleotide positions based on coding region are shown in the x-axis. The significant values for Tajima’s D was determined based on sample size. (TIFF) [file pcbi.1009801.s003.tiff]

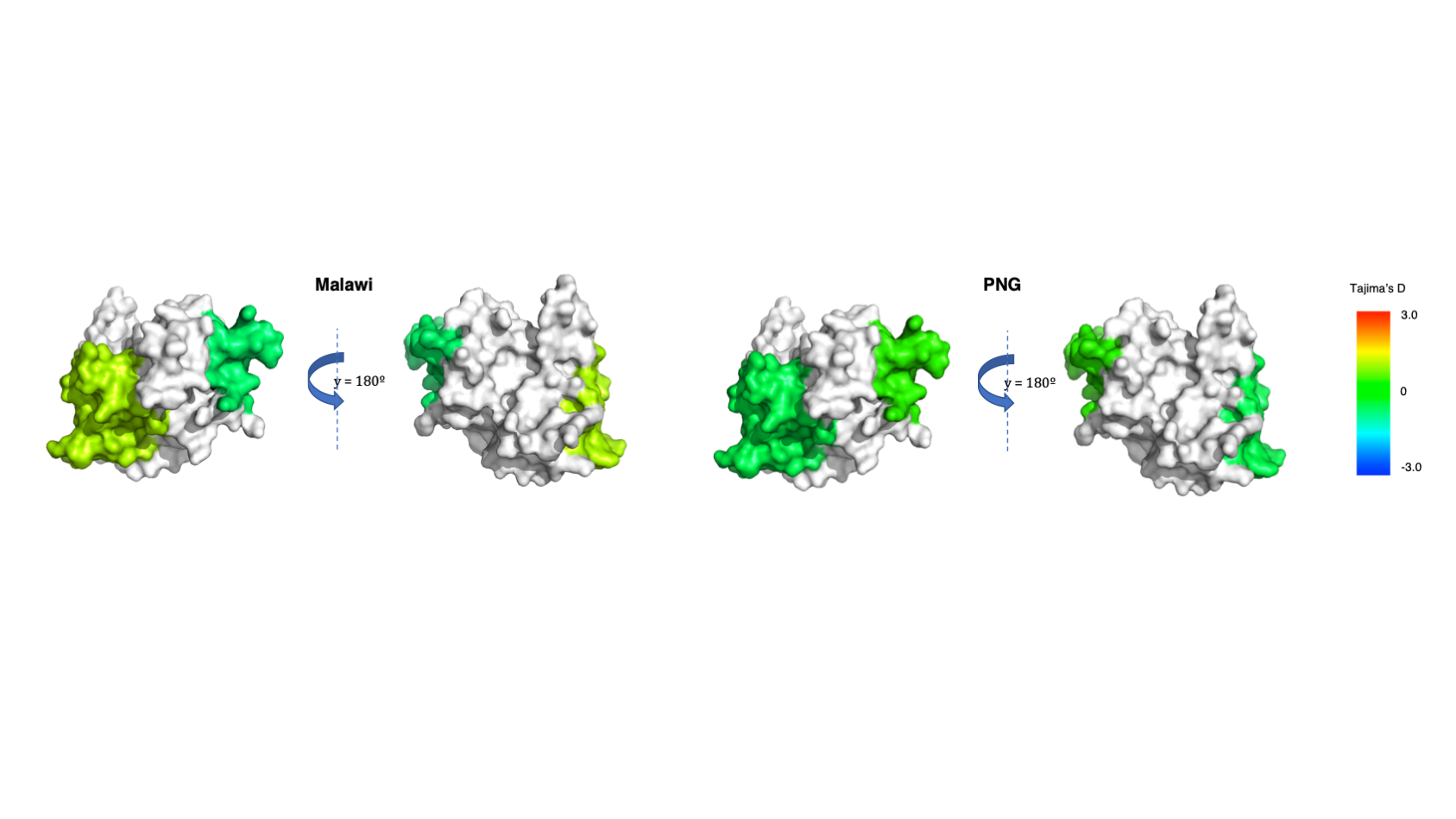

Supplement: S4 Fig — Tajima’s D (D*) calculation for geographic area or countries from Asia-Pacific and African regions for SERA5 (C-terminal) with incorporation of protein structural information using 15°A window. The structured region of SERA5 based on experimentally defined structure PDB code: 2WBF was used. The structure was coloured according to D* scores mapped to each residue with undefined D* were shown in grey. Only Malawi (n = 106), and PNG (n = 108) populations were shown. (TIFF) [file pcbi.1009801.s004.tiff]

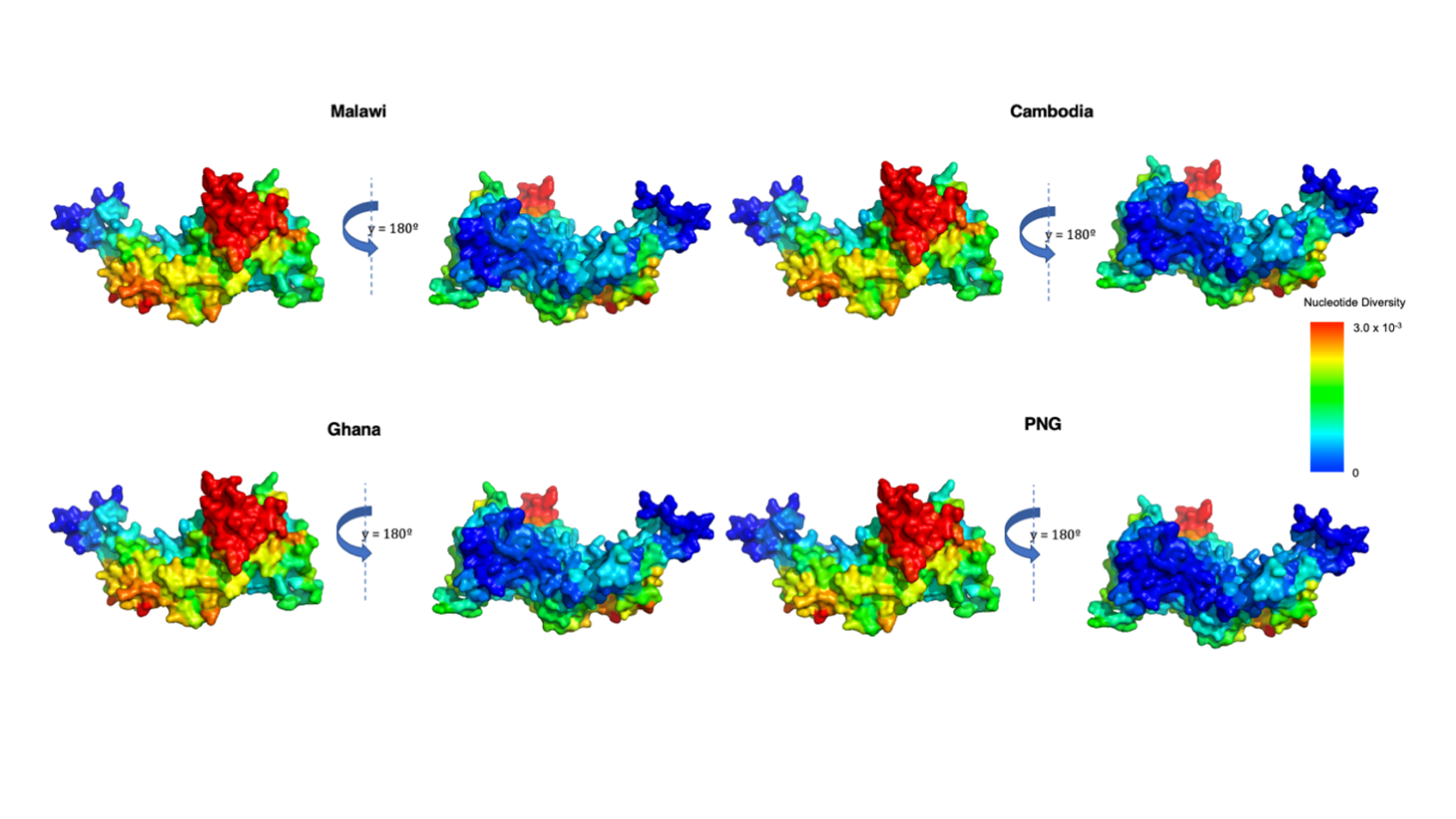

Supplement: S5 Fig — Nei’s nucleotide diversity calculation for geographic area or countries from Asia-Pacific and African regions for AMA1 with incorporation of protein structural information using 15°A window. Structure was coloured according to nucleotide diversity mapped to each residue. Sample size for each respective population are as follows: Malawi (n = 139), Ghana (n = 243), Cambodia (n = 433), and PNG (n = 112). Similar to selection pressure (determined by D*), silent face of AMA1 has low nucleotide diversity. (TIFF) [file pcbi.1009801.s005.tiff]

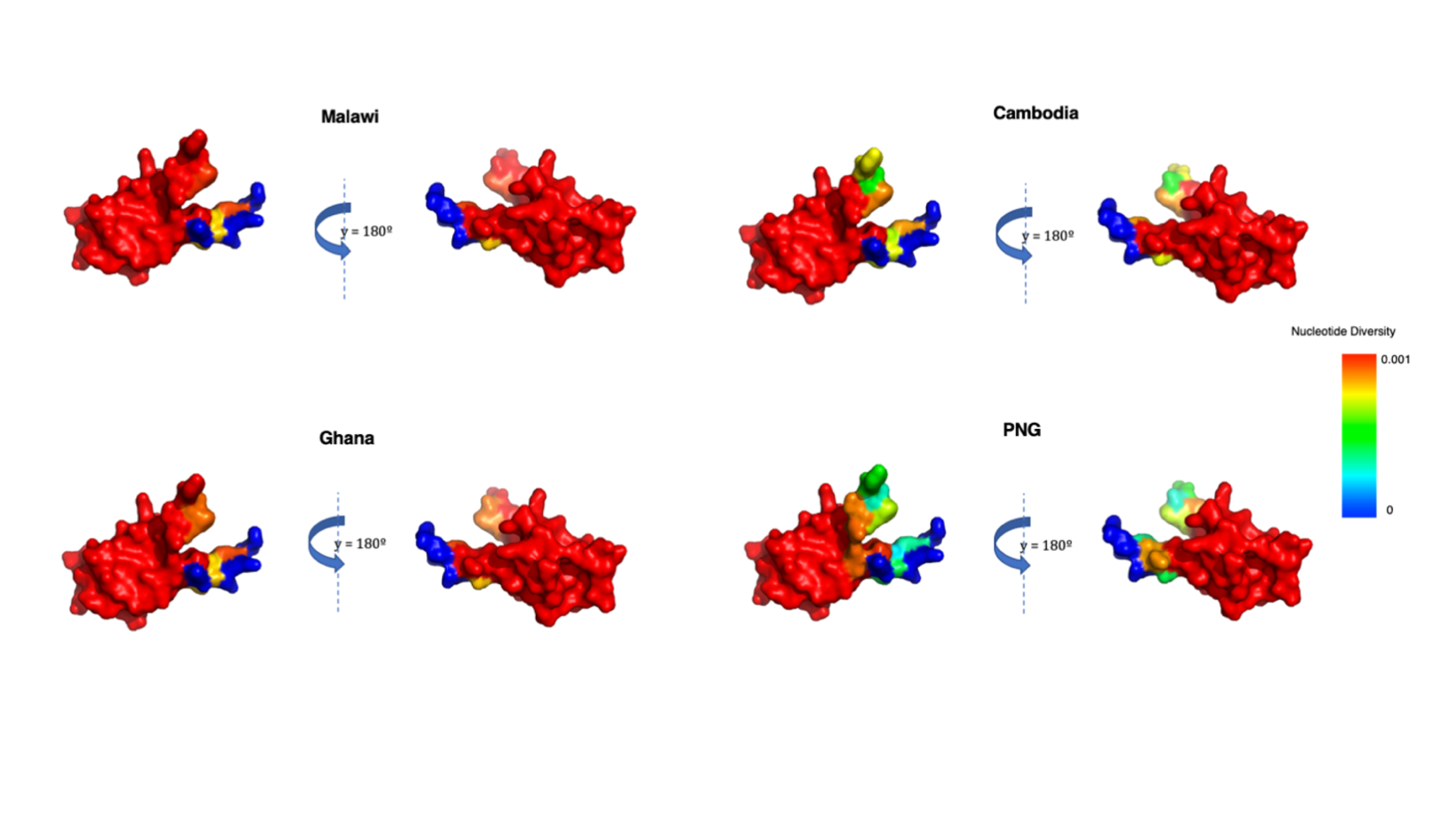

Supplement: S6 Fig — Nei’s nucleotide diversity calculation for geographic area or countries from Asia-Pacific and African regions for CSP (C-term) with incorporation of protein structural information using 15°A window. Structure was coloured according to nucleotide diversity mapped to each residue. Sample size for each respective population are as follows: Malawi (n = 135), Ghana (n = 223), Cambodia (n = 431), and PNG (n = 111). (TIFF) [file pcbi.1009801.s006.tiff]

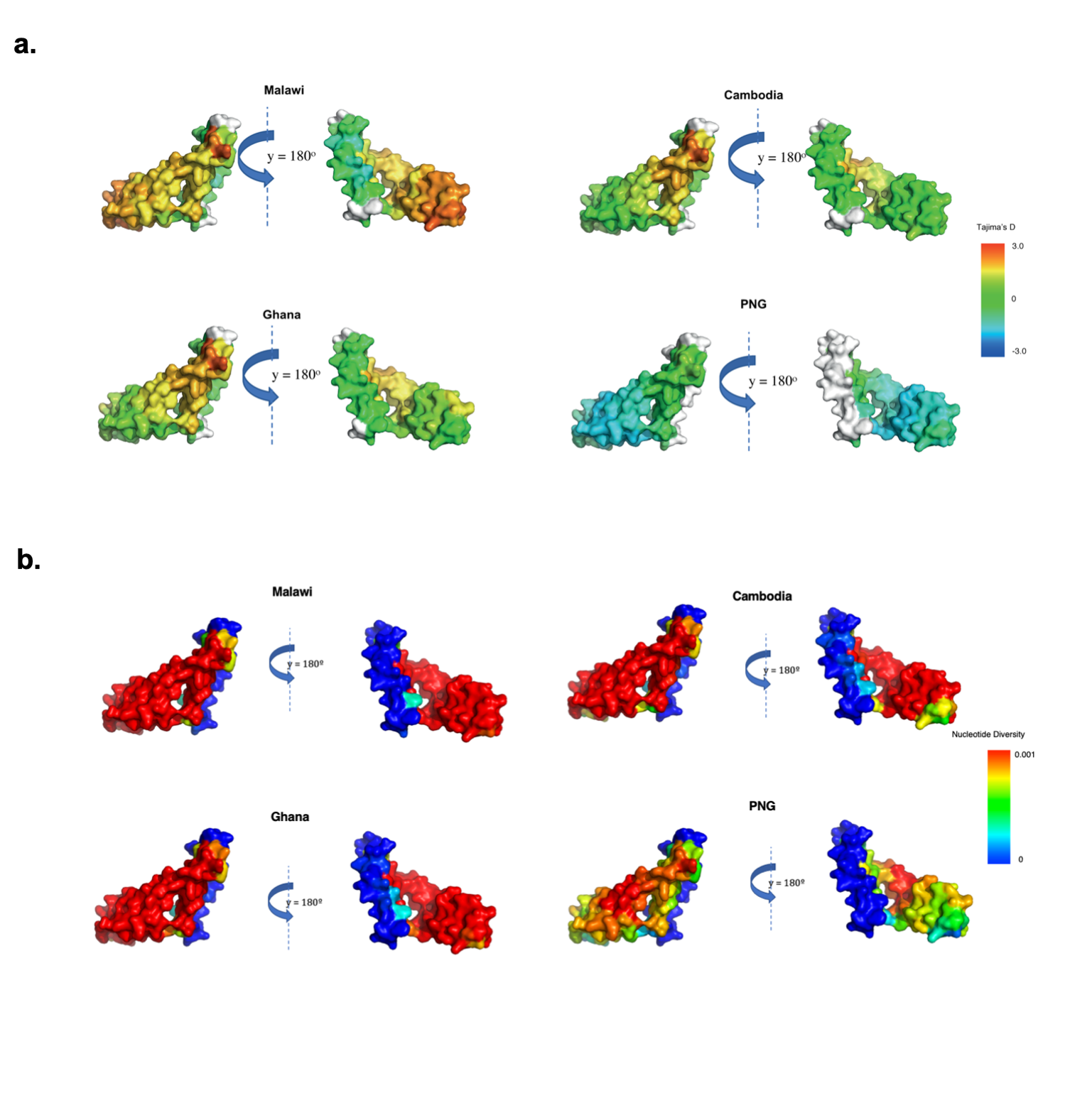

Supplement: S7 Fig — A. Tajima’s D (D*) calculations for populations from Asia-Pacific and African regions for CelTOS with incorporation of protein structural information using 15 Å window. Structure was coloured according to D* scores mapped to each residue with undefined D* were shown in white. 3D7-based ModPipe model of the P. vivax CelTOS based on 5TSZ template was used. Sample sizes: Malawi (n = 142), Ghana (n = 245), Cambodia (n = 433), and PNG (n = 112). B. Nei’s nucleotide diversity calculation for geographic area or countries from Asia-Pacific and African regions for CelTOS with incorporation of protein structural information using 15°A window. Structure was coloured according to nucleotide diversity mapped to each residue. Sample size for each respective population are as follows: Malawi (n = 142), Ghana (n = 245), Cambodia (n = 433), and PNG (n = 112). (TIFF) [file pcbi.1009801.s007.tiff]

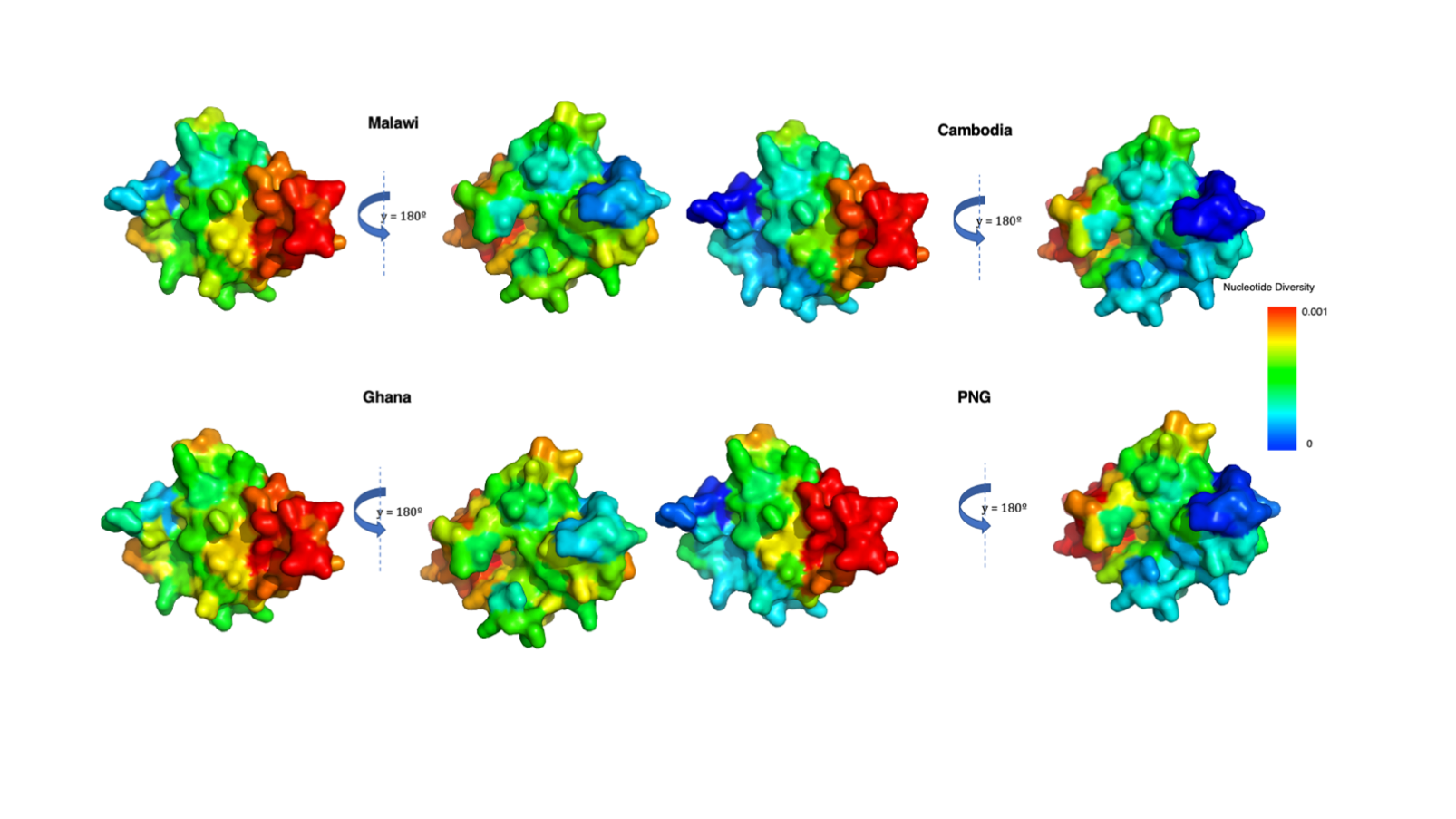

Supplement: S8 Fig — Nei’s nucleotide diversity calculation for geographic area or countries from Asia-Pacific and African regions for MSP1-19 with incorporation of protein structural information using 15°A window. Structure was coloured according to nucleotide diversity mapped to each residue. Sample size for each respective population are as follows: Malawi (n = 101), Ghana (n = 183), Cambodia (n = 270), and PNG(n = 72). (TIFF) [file pcbi.1009801.s008.tiff]

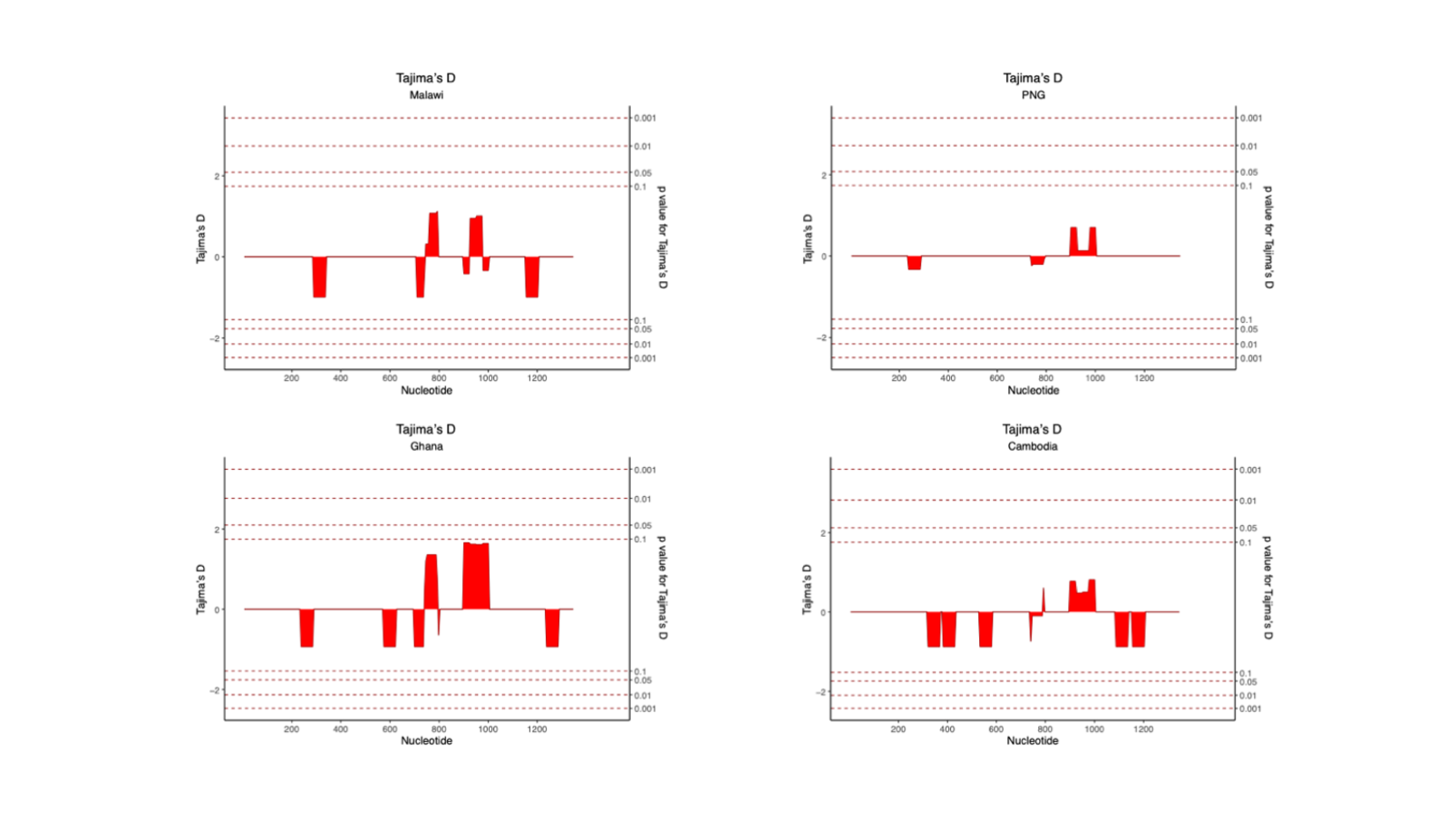

Supplement: S9 Fig — The sliding window analyses (a window size of 50 bp and a step size of 5 bp) calculated for Tajima’s D (D, red lines) for each population. Nucleotide positions based on coding region are shown in the x-axis. Significant value for Tajima’s D was determined by sample size. Sample size for each respective population are as follows: Malawi (n = 142), Ghana (n = 247), Cambodia (n = 433), and PNG (n = 112). (TIFF) [file pcbi.1009801.s009.tiff]

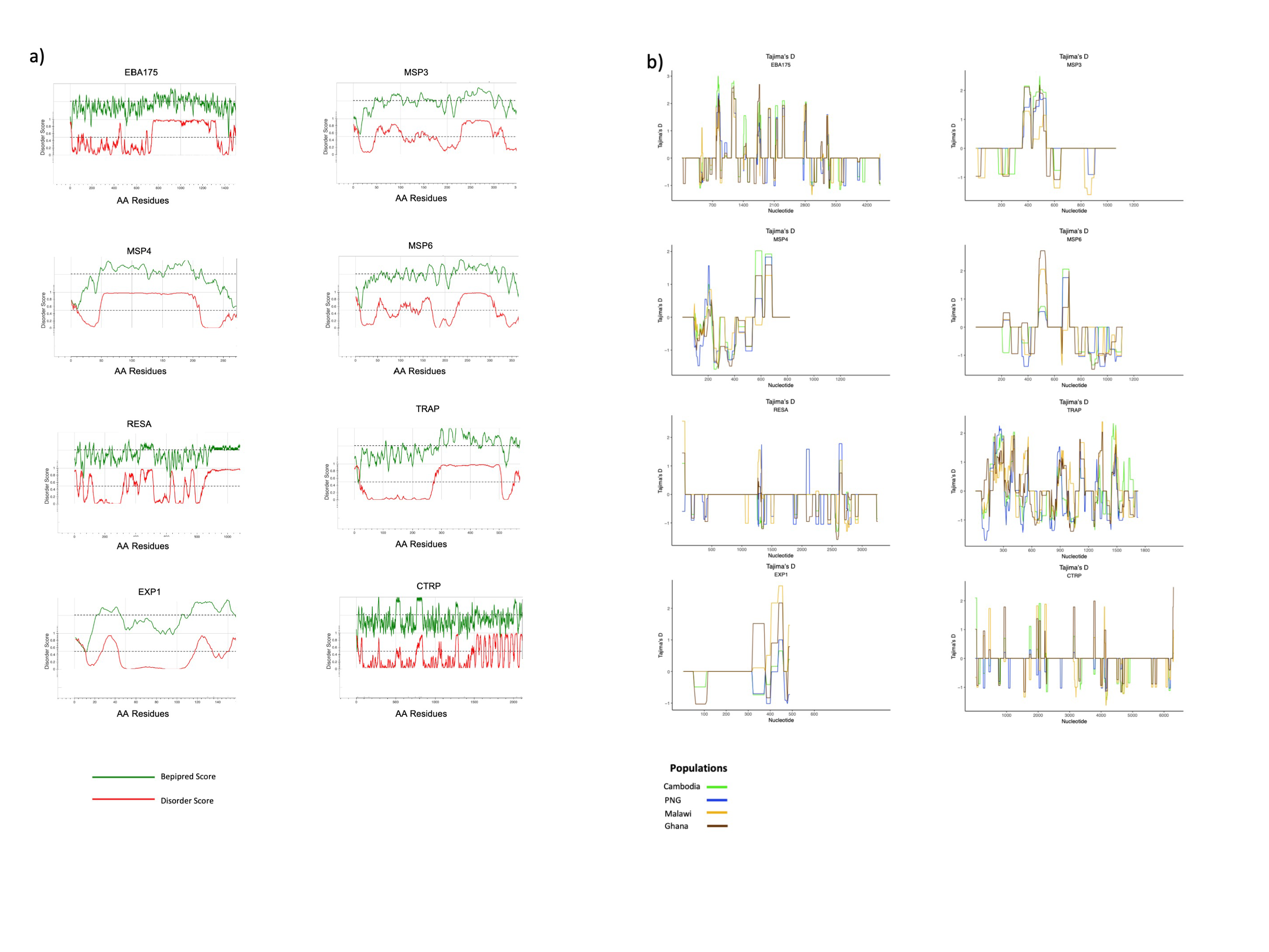

Supplement: S10 Fig — a) Computational predictions of protein disorder and B-cell epitopes in EBA175, MSP3, MSP4, MSP6, RESA, TRAP, EXP1 and CTRP. The green line represents the linear B-cell epitope mapping scores and the red line shows the protein disorder score, respectively. b) Tajima’s D statistics along the disordered antigens in samples from Cambodia, PNG, Malawi, and Ghana. It is calculated in the context of linear sequence level based on coding region with the sliding window approach (a window size of 50 bp and a step size of 5 bp). Nucleotide positions based on coding region are shown in the x-axis. Sample size for each respective population are as follows: Malawi (n = 106), Ghana (n = 208), Cambodia (n = 405), and PNG (n = 108). (TIFF) [file pcbi.1009801.s010.tiff]

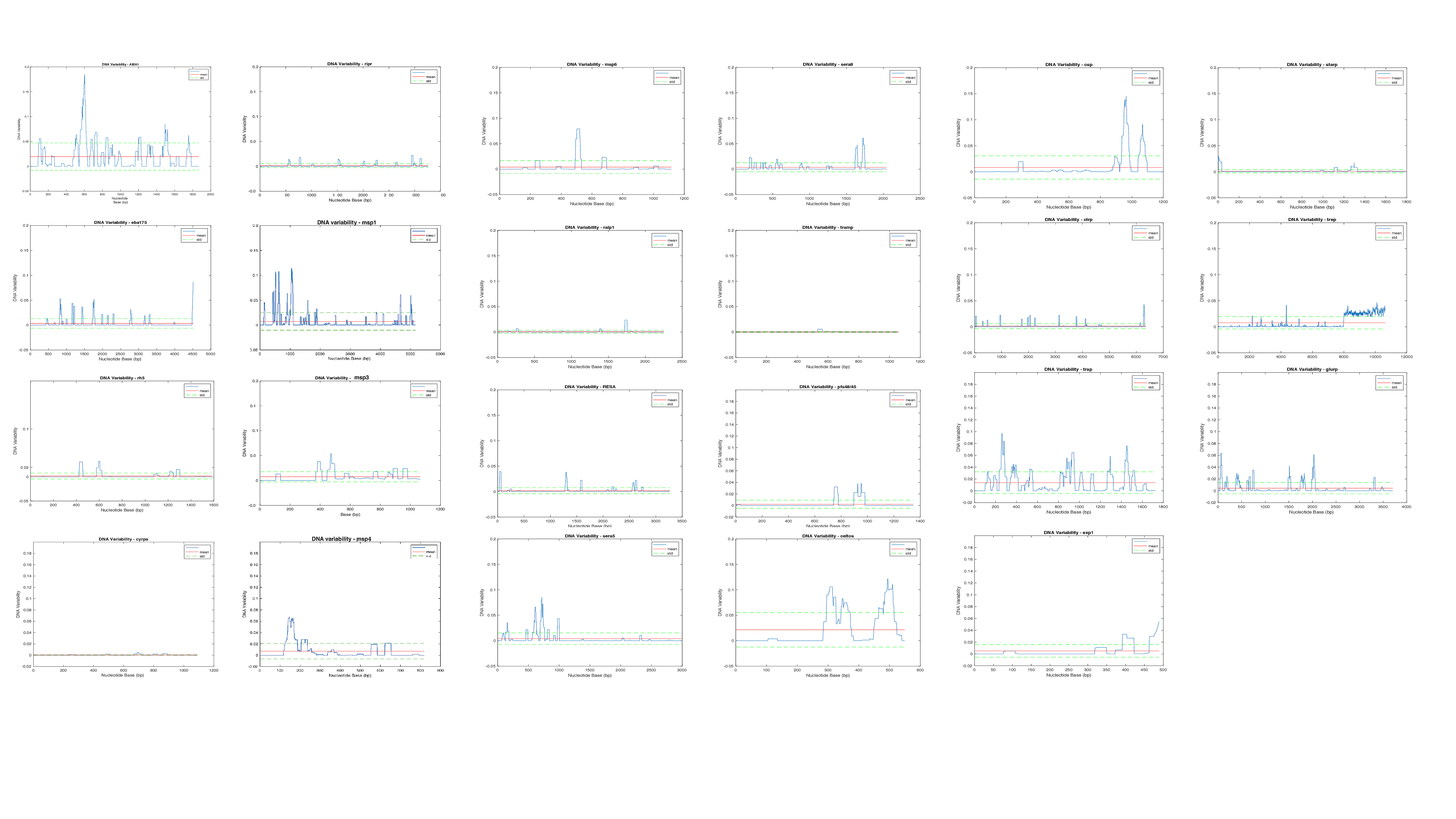

Supplement: S11 Fig — Sliding window analysis of sequence variability was calculated using algorithm from Proutski and Holmes et al., (1997) [1] implemented in MBEToolbox [2] using default parameters on MATLAB (version R2020a). Mean (red line), and standard deviation (green dotted line) within each antigen are shown. Nucleotide positions based on coding region are shown in the x-axis. (TIFF) [file pcbi.1009801.s011.tiff]
