# Supplementary material for: Global diversity and balancing selection of 23 leading Plasmodium falciparum candidate vaccine antigens
Source: PLoS Comput Biol. 2022 Feb 2;18(2):e1009801. doi: 10.1371/journal.pcbi.1009801 (PMC8843232; doi:10.1371/journal.pcbi.1009801)
Supplement: S1 Text — Table A: Antigen Diversity Summary (Full length or specific domain). Table B: Prevalence of haplotypes in dataset for analysed antigens and their proximity to 3D7 vaccine haplotype. (DOCX) [file pcbi.1009801.s012.docx]

**Supporting Materials**

***Table A:*** *Antigen Diversity Summary (Full length or specific domain) [1,2]*

| **Antigen Genes** | **Length (base pair)** | **Global NonSyn/Syn SNP Ratio (NS/SP)** | **Global Number of AA Haplotype** | **Global haplotype Diversity (Hd) (Median)** | **Global nucleotide Diversity (Median) (π * 10^-3)** | **Global 3D7 Proportion (%)** | **Sample Size (n)*** |
| --- | --- | --- | --- | --- | --- | --- | --- |
| *Csp* | 1194 | 3 | 152 | 0.9 | 4.43 | 1.03 | 1100 |
| *Csp (C-terminal)* | 249 | 31 | 110 | 0.91 | 19.32 | 2.97 | 1100 |
| *Ctrp* | 6345 | 2.8 | 212 | 0.78 | 0.66 | 0.07 | 1389 |
| *Trap* | 1725 | 23 | 563 | 0.98 | 7.06 | 0.41 | 1468 |
| *Trap (Ectodomain)* | 598 | 1.69 | 105 | 0.95 | 9.51 | 9.26 | 1468 |
| *Exp1* | 489 | 14 | 25 | 0.73 | 2.21 | 4.58 | 1445 |
| *Starp* | 1785 | 6 | 50 | 0.63 | 0.56 | 56.68 | 1452 |
| *Trep* | 10632 | 3.41 | 484 | 0.96 | 0.50 | 4.8 | 1453 |
| *Glurp* | 3702 | 4.04 | 383 | 0.98 | 2.44 | 0.83 | 1450 |
| *Ama1* | 1869 | 11.78 | 447 | 0.97 | 12.87 | 0.6 | 1478 |
| *Eba175 (FL)* | 4509 | 10.67 | 340 | 0.96 | 2.07 | 0 | 1477 |
| *Eba175 (RII)* | 1848 | 3.88 | 156 | 0.93 | 3.36 | 0.27 | 1477 |
| *Eba175 (RIII-V)* | 1477 | 0.91 | 82 | 0.57 | 1.07 | 34.3 | 1477 |
| *Rh5* | 1581 | 3.67 | 29 | 0.79 | 1.21 | 6.81 | 1499 |
| *Ripr* | 3261 | 5.12 | 108 | 0.91 | 0.78 | 22.56 | 1499 |
| *Cyrpa* | 1089 | 8 | 10 | 0.12 | 0.12 | 94 | 1485 |
| *Msp1* | 5163 | 6.16 | 603 | 0.98 | 4.3 | 0 | 1040 |
| *Msp1-19* | 277 | 3 | 12 | 0.64 | 5.98 | 23 | 1040 |
| *Msp3* | 1065 | 1.33 | 27 | 0.72 | 3.38 | 42.3 | 1202 |
| *Msp4* | 819 | 18.5 | 131 | 0.93 | 3.96 | 20.6 | 1358 |
| *Msp6* | 1116 | 22 | 44 | 0.75 | 2.59 | 39.9 | 1281 |
| *Ralp1* | 2250 | 2.86 | 26 | 0.52 | 0.30 | 55.3 | 1348 |
| *Resa* | 3258 | 3.36 | 145 | 0.93 | 0.95 | 1.13 | 1187 |
| *Sera5* | 2994 | 4.45 | 176 | 0.93 | 2.12 | 1.25 | 1356 |
| *Sera8* | 2040 | 9.5 | 198 | 0.93 | 1.73 | 0 | 1474 |
| *Tramp* | 1059 | 0.67 | 3 | 0 | 0.11 | 99.2 | 1491 |
| *Pfs48/45* | 1347 | 7 | 13 | 0.5 | 0.94 | 16.16 | 1480 |
| *Pfs48/45 (6C)* | 415 | 4 | 7 | 0.5 | 2.07 | 26.4 | 1480 |
| *Celtos* | 549 | 14.67 | 226 | 0.95 | 11.88 | 0.07 | 1488 |

*Different sample size amongst antigens due to further quality filtrations based on individual gene

***Table B:*** *Prevalence of haplotypes in dataset for analysed antigens and their proximity to 3D7 vaccine haplotype.*

| antigen | haplotype rank^*^ | Prevalence (%) | amino acid differences to 3D7 |
| --- | --- | --- | --- |
| CSP | 1 | 23.97 | 6 |
|  | 2 | 9.85 | 10 |
|  | 3 | 5.30 | 7 |
|  | 4 | 3.99 | 5 |
|  | 5 | 3.93 | 8 |
| CSP-C term | 1 | 24.19 | 5 |
|  | 2 | 9.86 | 9 |
|  | 3 | 5.31 | 6 |
|  | 4 | 4.69 | 8 |
|  | 5 | 4.00 | 4 |
| CTRP | 1 | 31.80 | 10 |
|  | 2 | 16.17 | 9 |
|  | 3 | 6.70 | 9 |
|  | 4 | 3.92 | 6 |
|  | 5 | 2.84 | 5 |
| TRAP | 1 | 6.74 | 12 |
|  | 2 | 3.88 | 17 |
|  | 3 | 3.74 | 13 |
|  | 4 | 2.72 | 15 |
|  | 5 | 2.38 | 11 |
| EXP1 | 1 | 39.69 | 2 |
|  | 2 | 16.33 | 1 |
|  | 3 | 9.43 | 1 |
|  | 4 | 9.15 | 2 |
|  | 5 | 6.76 | 2 |
| STARP | 1 | 56.71 | 0 |
|  | 2 | 20.85 | 1 |
|  | 3 | 3.99 | 2 |
|  | 4 | 3.58 | 1 |
|  | 5 | 3.30 | 1 |
| GLURP | 1 | 4.62 | 5 |
|  | 2 | 4.27 | 8 |
|  | 3 | 2.96 | 6 |
|  | 4 | 2.83 | 9 |
|  | 5 | 2.34 | 7 |
| TREP | 1 | 8.67 | 3 |
|  | 2 | 7.50 | 2 |
|  | 3 | 6.81 | 2 |
|  | 4 | 6.26 | 3 |
|  | 5 | 4.88 | 0 |
| AMA1 | 1 | 7.86 | 22 |
|  | 2 | 4.84 | 20 |
|  | 3 | 4.43 | 29 |
|  | 4 | 4.43 | 17 |
|  | 5 | 3.16 | 14 |
| EBA175 | 1 | 8.39 | 4 |
|  | 2 | 4.74 | 7 |
|  | 3 | 4.47 | 5 |
|  | 4 | 3.92 | 12 |
|  | 5 | 3.86 | 12 |
| EBA175 (RII) | 1 | 19.01 | 1 |
|  | 2 | 6.09 | 7 |
|  | 3 | 5.75 | 5 |
|  | 4 | 5.41 | 8 |
|  | 5 | 5.41 | 2 |
| RH5 | 1 | 36.49 | 1 |
|  | 2 | 15.08 | 2 |
|  | 3 | 7.67 | 4 |
|  | 4 | 7.01 | 1 |
|  | 5 | 6.81 | 0 |
| RIPR | 1 | 22.61 | 0 |
|  | 2 | 11.84 | 1 |
|  | 3 | 8.50 | 1 |
|  | 4 | 5.95 | 1 |
|  | 5 | 4.01 | 2 |
| CYRPA | 1 | 94.03 | 0 |
|  | 2 | 1.68 | 1 |
|  | 3 | 1.34 | 1 |
|  | 4 | 1.14 | 1 |
|  | 5 | 0.74 | 1 |
| MSP1 | 1 | 8.06 | 23 |
|  | 2 | 5.74 | 22 |
|  | 3 | 4.17 | 30 |
|  | 4 | 1.39 | 27 |
|  | 5 | 1.20 | 20 |
| MSP1-19 | 1 | 47.79 | 2 |
|  | 2 | 23.61 | 3 |
|  | 3 | 23.23 | 0 |
|  | 4 | 1.54 | 4 |
|  | 5 | 1.25 | 1 |
| MSP3 | 1 | 42.38 | 0 |
|  | 2 | 21.26 | 3 |
|  | 3 | 19.56 | 1 |
|  | 4 | 12.12 | 3 |
|  | 5 | 0.71 | 1 |
| MSP4 | 1 | 20.70 | 0 |
|  | 2 | 8.84 | 2 |
|  | 3 | 7.61 | 4 |
|  | 4 | 5.14 | 4 |
|  | 5 | 3.98 | 2 |
| MSP6 | 1 | 39.95 | 0 |
|  | 2 | 22.50 | 1 |
|  | 3 | 15.68 | 5 |
|  | 4 | 7.09 | 4 |
|  | 5 | 2.86 | 6 |
| RALP1 | 1 | 55.16 | 0 |
|  | 2 | 32.87 | 1 |
|  | 3 | 4.15 | 1 |
|  | 4 | 2.35 | 1 |
|  | 5 | 1.11 | 1 |
| RESA | 1 | 24.10 | 3 |
|  | 2 | 9.14 | 2 |
|  | 3 | 4.64 | 2 |
|  | 4 | 4.36 | 3 |
|  | 5 | 3.80 | 4 |
| SERA5 | 1 | 11.50 | 13 |
|  | 2 | 8.03 | 8 |
|  | 3 | 7.66 | 14 |
|  | 4 | 7.22 | 9 |
|  | 5 | 6.85 | 12 |
| SERA8 | 1 | 11.65 | 4 |
|  | 2 | 9.78 | 4 |
|  | 3 | 6.77 | 6 |
|  | 4 | 6.03 | 3 |
|  | 5 | 5.22 | 6 |
| TRAMP | 1 | 99.20 | 0 |
|  | 2 | 0.67 | 1 |
|  | 3 | 0.13 | 1 |
| Pfs48/45 | 1 | 54.01 | 3 |
|  | 2 | 16.16 | 0 |
|  | 3 | 15.75 | 1 |
|  | 4 | 9.15 | 2 |
|  | 5 | 1.80 | 2 |
| CelTOS | 1 | 11.90 | 9 |
|  | 2 | 7.42 | 7 |
|  | 3 | 6.35 | 6 |
|  | 4 | 4.81 | 7 |
|  | 5 | 4.41 | 10 |

* Only the most common 5 haplotypes are shown in the table for most antigens except TRAMP which has only 3 haplotypes.

**Geographically variable selection for Pfs48/45**

Consistently, moderately *D* scores (1.5 – 2) were observed in the region corresponding to nucleotide residues 780 – 800 in most of the populations (Fig S9). In addition, nucleotide residues 900 – 100 were also under balancing selection within African populations (moderately high *D* of 1.7) (Fig S9). Of these, corresponding amino acid residues N299 and N303 are known to be involved in N-linked glycosylation [3]. Patterns of selection were varied amongst observed populations. However, due to limited number of Pfs48/45 non-synonymous polymorphisms driving these patterns, the results should be interpreted with care.

**Supporting References**

1. Proutski, V. and Holmes, E., 1998. SWAN: sliding window analysis of nucleotide sequence variability. Bioinformatics, 14(5), pp.467-468.

2. Cai, J., Smith, D., Xia, X. and Yuen, K., 2006. MBEToolbox 2.0: An enhanced version of a MATLAB toolbox for Molecular Biology and Evolution. Evolutionary Bioinformatics, 2, p.117693430600200.

3. Kundu P, Semesi A, Jore MM, Morin MJ, Price VL, Liang A, et al. Structural delineation of potent transmission-blocking epitope I on malaria antigen Pfs48/45. Nat Commun. 2018;9: 1–9. doi:10.1038/s41467-018-06742-9
